# Dissociable species-specific impact of Aβ on static and dynamic functional connectomes

**DOI:** 10.64898/2026.04.26.720907

**Published:** 2026-04-29

**Authors:** Matteo M. Grudny, Nicholas Rodriguez, Thomas J. Murdy, Zachary D. Simon, Quan Vo, Wen Li, Matthew R. Burns, Damon G. Lamb, Catherine C. Kaczorowski, Paramita Chakrabarty, Marcelo Febo

**Affiliations:** 1 Department of Psychiatry, University of Florida, Gainesville, FL; 2 Department of Neuroscience, University of Florida, Gainesville, FL; 3 Department of Neurology, University of Florida, Gainesville, FL; 4 McKnight Brain Institute, University of Florida, Gainesville, FL; 5 Advanced Magnetic Resonance Imaging and Spectroscopy Facility, University of Florida, Gainesville, FL; 6 Center for Translational Research on Neurodegenerative Diseases, University of Florida, Gainesville, FL; 7 Normal Fixel Institute for Movement Disorders, University of Florida, Gainesville, FL; 8 Program in Medicine, Tufts University School of Medicine, Boston, MA; 9 Department of Neurology, University of Michigan School of Medicine, Ann Arbor, MI; 10 Brain Rehabilitation Research Center, Malcolm Randall VA Medical Center, Gainesville, FL

## Abstract

Temporal dynamics in functional connectomes offer a physiologically grounded signature of ‘hidden’ pathologies during preclinical stages of Alzheimer’s disease (AD). We evaluated the effect of beta-amyloid (Aβ) on dynamic functional connectomes in transgenic mice and human subjects. Functional magnetic resonance images (fMRI) were collected in two strains of Aβ mice. fMRI-derived connectomes were segmented into discrete states using a hidden Markov model and network strength, efficiency, and transitivity were analyzed per state. Human fMRI-derived connectome measures were analyzed across 3 states. Static network measures were significantly different between Aβ mice and controls, the former having high values for strength, efficiency and clustering coefficient in anterior cingulate, hippocampus and retrosplenium. Dynamic network measures were stable within-states in Aβ mice. Similarly, human subjects with high Aβ had high node strength in precuneus and temporoparietal areas compared to low Aβ. In contrast, however, high Aβ was associated with high state switch rates, high fractional occupancy and state dwell times. Also, global strength, efficiency, and transitivity were less stable within states in the high Aβ group. Our results indicate that static, but not dynamic, connectome strength, efficiency and network integration are increased in Aβ mice, while dynamic network states appear less stable in human functional connectomes. This data supports a dissociable, species-specific impact of Aβ, with dynamic network alterations present in humans but not in Aβ mouse models, suggesting additional non-Aβ-driven influences on dynamic functional connectivity in preclinical AD.

## Introduction

The expression of core Alzheimer’s disease (AD) symptoms is preceded by a protracted period in which known pathological hallmarks act alongside ‘latent’ pathologies to progressively impair functional networks serving cognition[1, 2]. During this asymptomatic prodromal phase extracellular beta amyloid (Aβ) and intraneuronal tau steadily accumulate, deteriorating brain wide neural circuits serving memory, emotion, and motor functions[1]. Network-centric functional magnetic resonance imaging (fMRI) has uncovered cognition-related brain regional interactions adversely affected by Aβ, tau, with several imaging studies supporting ‘hidden’ pathological network states during this period[3–7]. Understanding how functional brain networks are reconfigured during preclinical AD could uncover novel pathological events to mark early-stage disease vulnerability[8].

Elevated Aβ is associated with altered functional connectivity in medial frontal, posterior cingulate, temporal-parietal and precuneus areas of the default mode network (DMN), and parahippocampal regions [9–11]. Functional connectivity between precuneus and anterior hippocampus in individuals with high Aβ levels is increased, while a decline in functional connectivity is observed in normal aging individuals with low Aβ [9]. Significant declines in functional connectivity across DMN areas are also reported in AD and mild cognitive impairment (MCI)[7, 12, 13], although such reductions are linked to tauopathy and neuronal atrophy[14–18]. Similarly, neuroimaging studies using transgenic rodents with human familial mutations of the Aβ protein precursor (AβPP) support a link between Aβ and increased functional connectivity involving frontal, cingulate, hippocampal and DMN-like regions[19, 20], while other AβPP strains have age-progressive reductions in functional connectivity in parietal, motor, retrosplenial and dorsal hippocampal regions[21–23], particularly in the presence of tau[23]. Aβ plaque burden in mice is associated with reduced functional connectivity in anterior cingulate while increases are observed in pre-plaque stages[24], a result linked to rising Aβ levels[19, 25]. Neuroimaging findings thus suggest pathology-specific changes in functional connectivity that are of considerable value to further uncover, especially during prodromal AD[15].

Apart from interregional weakening or strengthening of cortical functional connectivity with Aβ[9–11], network topology changes are also reported[26, 27]. The reconfiguration of functional interactions between network nodes is key to understanding progression to AD[27]. Alterations in network transitivity (a global version of node clustering coefficient) and average shortest path lengths, which are two graph theory metrics providing insight into network integration and efficiency, are reported in AD and MCI[28–32]. Other studies have observed AD-associated changes in modular organization[33], rich club organization[34], and altered ‘hubness’ based on eigenvector centrality[35], the latter also observed in AβPP-PS1 mice [29, 31, 32]. Amyloid positivity has been closely linked to node strength, clustering coefficient, and efficiency, although this has varied across several studies[36–38]. While rodent models have been used extensively to explore the role of Aβ on resting state networks, few have investigated the relationship between Aβ and functional connectome measures[36–38]. In addition, there is growing evidence that Aβ and other AD pathologies affect functional connectivity network measures across distinctly discernible temporal states[39–43]. The temporal evolution of functional connectivity during a single session of imaging is thought to capture physiologically relevant dynamic states[44, 45], perhaps internally driven by variability in cognitive and emotional neuronal circuit processing [46, 47]. Risk of AD and dementia, and progression to AD, are associated with temporal variability in functional connectivity[43, 48, 49]. Temporal variability in network connectivity is also observed in subjective cognitive decline, with pairwise regional changes in dynamic connectivity that are specific to cognitive decline versus depression [50]. Indeed, dynamic networks have been studied in rodents and compared to human and non-human primates, with emerging features in common[51].

In the present study, we used a variant of the hidden Markov model (HMM)[52] to inference ‘hidden states’ underlying spatiotemporal functional connectome measures of integration and segregation in mice bearing Aβ, and in a group of ADNI data sets of elderly individuals with low versus high Aβ and no cognitive impairment. Both in mouse and in human data sets, Aβ is associated with high network strength, efficiency and clustering coefficient in DMN areas, and DMN-like areas in mice. However, while in mice we find that the effect of Aβ on these network measures persists stably within states, in humans we observe that high Aβ is linked to variable strength, efficiency and clustering coefficient within states. This cross-species difference suggests ‘hidden’ pathological mechanisms in human AD that are not present in mice only harboring Aβ pathology.

## Materials and methods

### Mice

This study used two strains of adult transgenic (TG) mice that develop amyloid plaques, TgCRND8 mice[53] and a sub-strain of the recombinant inbred AD-BXD line[54]. Age, strain, and sex-matched non-transgenic (NTG) mice served as controls. Mice were housed in groups of 3–4 in a temperature- and humidity-controlled room, inside conventional air filtered cages (dimensions: 29 × 18 × 13 cm) with food and water available *ad-libitum* (vivarium lights on from 07:00–19:00 h).

Methods describing the development and maintenance of TG strains are published[54–56]. TgCRND8 mice have early onset expression of human mutant beta-amyloid protein precursor (AβPP) (Swedish AβPP KM670/671NL and Indiana AβPP V717F), which increases human AβPP 5-times above endogenous murine AβPP. AD-BXD mice (referred to here as 5XFAD) are hemizygous for the dominant 5XFAD transgene[57], which consists of 5 human mutations known to cause familial AD (three AβPP mutations: Swedish K670N, M671L, Florida I716V, and London V717I and two presenilin-1 PSEN1 mutations: M146L and L286V).

A total of 95 mice were used, including 7–8 month old (m) (young NTGY, n=19; 7 female mice), 15–19 m (middle age NTGM, n =9; 5 female mice), 21–23 m (aged NTGA, n = 25; 10 female mice) NTG mice, 6–9 m (young CRND8Y, n=13; 7 female mice) and 16–18 m (middle age CRND8M, n = 5; 3 female mice) CRND8 mice, and 7–8 m (young 5XFADY, n= 12; 5 female mice) and 15–18 m (middle age 5XFADM, n=12; 5 female mice) 5XFAD mice. TG mice were scanned at two timepoints, with TgCRND8 losing 8 mice by 16 m and 5XFAD mice showing 100% survival at 15 m. Littermates for the 5XFAD mice included NTG C57BL6/J (B6) and B6D2 (B6xDBA/2J F1). The background strain for TgCRND8 was B6C3H (B6xC3H/HeJ F1) obtained from the Jackson laboratory (Bar Harbor). Procedures were approved by the Institutional Animal Care and Use Committee of the University of Florida and follow all applicable NIH guidelines.

### Magnetic resonance imaging

Data were collected on an 11.1 Tesla MRI system equipped with Resonance Research Inc. gradients (RRI BFG-240/120-S6, 1000 mT/m at 325 Amps, 200 μs risetime) and a Bruker AV3 HD console running Paravision 6.0.1. A custom in-house quadrature transceive surface radiofrequency (RF) coil was used for image acquisition (Advanced Magnetic Resonance Imaging and Spectroscopy Facility, Gainesville, FL). Mice were imaged sedated under 0.1 mg/kg (i.p.) dexmedetomidine and a continuous paranasal flow of 0.25 % isoflurane (0.5 L/min flow; 70% nitrogen and 30% oxygen; Airgas, Inc.). An infusion line subcutaneously delivered supplemental dexmedetomidine during scanning (0.1 mg/kg/ml at an infusion rate of 25 μl/hour (PHD-Ultra microinfusing pump, Harvard Apparatus, Holliston, MA). Functional MRI scans were collected at least 50 minutes after the dexmedetomidine injection. Mice were kept warm during scanning and spontaneous breathing rate was monitored.

A T2-weighted Rapid Acquisition with Refocused Echoes (RARE) sequence was acquired with the following parameters: echo time (TE) = 41 ms, repetition time (TR) = 4 seconds, RARE factor = 16, number of averages = 12, field of view (FOV) of 15 mm × 15 mm and 0.9 mm thick slice, and a data matrix of 256 × 256 (0.06 mm^2^ in plane) and 14 interleaved ascending coronal (axial) slices, covering the entire brain from the rostral-most extent of the anterior prefrontal cortical surface, caudally towards the upper brainstem and cerebellum. Functional images were collected using a single-shot spin echo planar imaging (EPI) sequence with the following parameters: TE = 15 ms, TR = 2 seconds, 600 repetitions, FOV = 15 × 15 mm and 0.9 mm thick slice, and a data matrix of 64 × 48 (0.23 × 0.31 mm in plane) with 14 interleaved ascending coronal slices in the same position as the anatomical scan. Ten dummy EPI scans were run prior to acquiring data. Respiratory rates, isoflurane and dexmedetomidine delivery, temperature, lighting, and room conditions were kept constant across subjects.

### Image processing

Images were processed using Analysis of Functional NeuroImages (AFNI)[58], FMRIB Software Library (FSL)[59], and custom scripts written in MATLAB[60]. Voxel time series were ‘despiked’, motion-corrected, and drift-corrected. We ran independent components analysis (ICA)[61] to evaluate time series and generate nuisance regressors. These nuisance signals were identified by the presence of scattered or individual voxels with large irregular and spurious temporal shifts, high frequency and/or pulsatile temporal patterns, mostly localized to clusters of voxels along brain edges and spurious voxels in white matter areas and ventricles. A list of nuisance regressors was generated per mouse. fMRI scans were ‘denoised’ using these regressors, band pass filtered to suppress frequencies outside of a bandwidth of 0.009 Hz to 0.2 Hz and spatially filtered. This frequency bandwidth allowed a broad range of signal features to explore dynamic network states (see below). A functional image volume was used for multi-subject fMRI template-construction using Advanced Normalization Tools (ANTs)[62]. The single fMRI volume per subject was bias field corrected, and brain extracted before template construction. Affine and nonlinear transformations were applied to preprocessed fMRI scans, and these were analyzed in template space. Inverse linear transform of a mouse atlas[63] parcellation to the multisubject template was performed to create 51 regions of interest (ROI) masks per hemisphere (102 total bilateral ROI masks) to extract time series.

### Static networks

Pearson correlations between pairs of ROIs (totaling ~102^2^) were carried out in MATLAB. The r coefficients were Fisher z transformed, organized into weighted undirected symmetrized matrices, and graph calculations for a network density of 15% carried out using Brain Connectivity toolbox[64]. These included network strength, characteristic path length, global efficiency, assortativity, modularity and transitivity[60]. These graph measures were chosen to evaluate global functional network integration, segregation, and efficiency, that are affected in Alzheimer’s disease[12] and in amyloid mouse models[37]. *Node strength* was calculated as the sum of edges (Pearson r’s) per ROI, with the global average across ROIs used as an indicator of network strength. *Characteristic path length* was calculated by first converting correlation matrices to length matrices and then using Dijkstra’s algorithm to estimate distances. Thus, characteristic path length was the mean weighted distances across all nodes and *global efficiency* was the inverse distance matrix. For *transitivity*, correlation matrices were scaled to 0,1 and the ratio of triadic groups (strongly interconnected groups of 3 nodes) normalized to all possible triplets was used as a measure of how well integrated nodes are within the network. Node level versions for transitivity (clustering coefficient) and global efficiency (node efficiency) were also calculated. *Assortativity* was calculated as the correlation between strength values of above-diagonal row and column elements of adjacency matrices. *Modularity* was calculated using the classical optimal community structure algorithm that indexes the degree of maximum within-group and minimal between-group edges. This was used as an index of segregation of nodes into functional communities. Matrices were averaged within experiment groups, mean matrices set to an edge density of 15%, node strengths calculated and then visualized in BrainNet Viewer with nodal spheres overlaid onto a mouse atlas 3-dimenstional translucent shell representing node strength and the interconnecting lines the above-threshold edge weights, as previously described[60].

### Dynamic networks

We used the MATLAB version of the hidden Markov model multivariate autoregressive (HMM-MAR) toolbox, published by Vidaurre[65], to segment fMRI time series into quasi-stationary network states[52]. Using this approach, we evaluated the effect of age and Aβ on dynamic functional connectivity networks in mice. Formal description of HMMs applied to fMRI time series has been described [52]. Briefly, an HMM segments multivariate time series into a sequence of discrete “states” with Markov property. For a consecutive series of observations, $X_{1:T}$, and hidden states, $Z_{1:T}\in \{1,\ldots ,K\}$, with joint distribution $p(X,Z|\theta )=p\left(Z_{1}|\pi \right)\prod_{t=2}^{T}p\left(Z_{t}|Z_{t-1},A\right)\prod_{t=1}^{T}p\left(X_{t}|Z_{t},\Phi \right)$[66], the model parameters are denoted by the term θ={π,A,Φ}, where π is the initial state distribution, A is the K×K transition matrix with row sums equal to 1, and Φ are the emission parameters[66]. Here we used Gaussian emissions with MAR order 0 (no autoregression; thus, Φ = one mean and covariance per state) after standardization (z-scoring) and dimensionality reduction (PCA) of windowed connectivity features. Inference is performed using variational Bayes, as implemented in HMM-MAR, which maximizes the variational free energy (evidence lower bound)[66]. A Dirichlet prior (controlled by the DirichletDiag parameter in HMM-MAR, which we set to 100[52]) is placed on each row of A for reasonable self-transitions. This yields posterior state probabilities per time point, Gamma, $\Gamma_{t,k}=p\left(Z_{t}=K|X,\theta \right)$, and the most probable state sequence via the Viterbi algorithm. Representative ‘state mean networks’ for each HMM state was estimated by Γ-weighted averaging of connectivity windows that were mapped back to ROI×ROI matrices. For each state K, we computed a mean edge vector, $M_{K}=\left(\sum_{t}\Gamma_{t,K}\cdot X_{t}\right)/\left(\sum_{t}\Gamma_{t,K}\right)$, where $X_{t}$ is the vectorized upper-triangle of the windowed Fisher-z connectivity and $\Gamma_{L,K}$ is the posterior probability of state K at time t. We then filled $M_{K}$ into the upper triangle, symmetrized, and set the diagonal to zero to obtain the state’s mean adjacency, G^K^. We derived dynamic features (fractional occupancy, mean dwell time, and the empirical transition matrix) from Γ and the corresponding most probable state (MAP/Viterbi) sequence. Graph-theoretic measures per state were computed on the state mean adjacencies, G^K^, after proportional thresholding to a 15% graph density.

**Fig 1.**
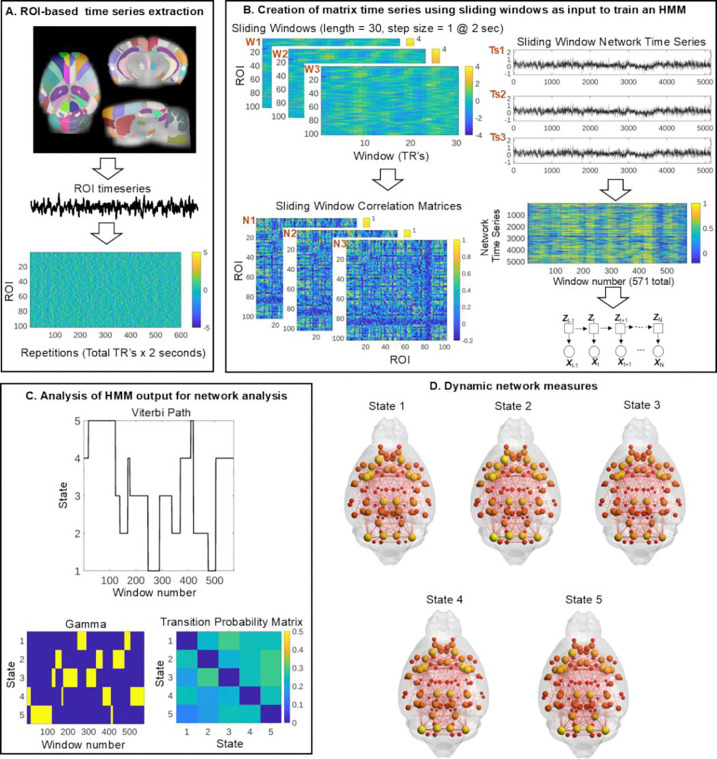
Processing of mouse fMRI signals for HMM-MAR. A) Signals were extracted from post-processed images aligned to a mouse brain parcellation[63]. B) Following a sliding window, network matrices were constructed per window and vectorized and organized in a network x window matrix as input to HMM-MAR[75]. C) HMM outputs. D) Network visualizations across states.

The schematic in Figure 1 summarizes the preprocessing steps in HMM-MAR. We imported ROI time series into MATLAB (600 time points by 102 ROIs) and applied a sliding window (30 TR, 1 TR step, so 60-second segments with 2 second stride) to generate 571 windows ([timepoints – windows] / step size + 1). For each window we computed pairwise correlations, Fisher z-transformed them, and vectorized the upper triangle ([102 × 101]/2] for a total of 5,151 edges. The result is a 571 × 5,151 window-by-edge matrix per subject (later concatenated across subjects) as input to HMM-MAR. We fit a Gaussian HMM with shared full covariance across states, standardization, and PCA (retaining ~52% explained variance). We used 15 restarts (initrep) with 25 initialization cycles (initcyc) and allowed up to 1,000 inference cycles. We swept K = 3–10 (in line with prior rodent work[51, 67, 68], although we note other reports inference a greater number of states[69, 70]) and selected K=5. At higher K (6–10) increasingly many ‘microstates’ with negligible durations (<1–2 TR) were observed. From these dynamic states, we analyzed fractional occupancy (FO: fraction of total time spent in each state) and dwell windows (number of windows per each state) as measures of dynamic functional connectivity variability.

### Histology

Aβ plaques were quantified in hemi-brains of TgCRND8 mice (n=5; 6m mice n = 3, 12m mice n = 2 mice). Brain harvesting was done at the University of Florida and tissue preservation, processing, clearing, immunolabeling and volumetric imaging of immunolabeling, and image post-processing was carried out at LifeCanvas Technologies (Cambridge, MA). Paraformaldehyde-fixed brains were preserved using SHIELD reagents[71], delipidated and labeled using eFLASH[72] technology on a SmartBatch+ device. Aβ plaques were co-labeled with propidium iodide (PI) nuclear stain as background. Immunolabeling was carried out with Aβ anti-body (mouse IgG2a, MCA-AB9, Encor) and donkey anti-mouse Alex Fluor 488 secondary. After immunolabeling, samples were incubated in EasyIndex (RI = 1.52) for refractive index matching. Intact whole brain volumes were imaged using a SmartSPIM axially swept light sheet microscope using a 3.6x field of view (0.2 NA objective) at 20 fps during volumetric acquisition with 488 and 561 nm laser lines for Aβ and PI. Registration to the mouse common coordinate framework v3 (Allen Mouse Brain Atlas) followed published pipelines[73], SmartAnalytics code was used for Aβ quantification and image processing [73] and analysis of percent Aβ occupation per ROI[74] (LifeCanvas Technologies, Cambridge, MA).

### Static and dynamic functional networks in low and high amyloid density

We analyzed static and dynamic functional networks in an Alzheimer’s disease neuroimaging initiative (ADNI3) dataset (approved under University of Florida protocol #: NH00047739). Out of 34 scans, 27 were controls and 7 had subjective memory complaints. None were diagnosed with mild cognitive impairment or Alzheimer’s. The dataset included 16 subjects with high amyloid density (age 72.2 ± 7.0; 2 males; 1.41 ± 0.19 AV45 SUVR and 1.38 ± 0.17 at baseline) and 18 with low amyloid density, defined by AV45 normalized binding (age 69.2 ± 6.2; 2 males; 1.00 ± 0.03 AV45 SUVR and 1.01 ± 0.04 at baseline)(t-test: low vs high Aβ, *p* = 4.4 × 10^−7^). Low and high Aβ groups all had CDRSB scores of 0 with no evidence of cognitive impairment from their Montreal Cognitive Assessment scores (MoCA: low Aβ = 27.2 ± 1.69, high Aβ = 25.6 ± 2.9; t-test *p* = 0.011), mini-mental state exam scores (MMSE: low Aβ = 29.38 ± 0.69, high Aβ = 28.5 ± 1.55; t-test *p* = 0.04), and Alzheimer’s dementia assessment scale scores (ADAS-Cog11: low Aβ = 5.79 ± 2.1, high Aβ = 6.4 ± 4.2; t-test *p* = 0.6; ADAS-Cog13: low Aβ = 8.62 ± 3.2, high Aβ = 9.85 ± 6.1; t-test *p* = 0.48).

Axial EPI scans were collected on 3 Tesla Siemens scanners (70% Prisma, 21% Verio, and 9% Skyra) with the following parameters: TE=30ms, TR=3seconds, dimensions 3.4 mm^3^, matrix size = 64×64×48 voxels, 197 repetitions (10-minute scan duration). Subjects had eyes open during fMRI scanning. The first 7 volumes in the 197 volume time series were removed prior to processing steps. The steps included time series spike removal, slice timing correction, motion and drift correction. ICA based identification of ‘noisy’ time series voxels was used to regress these prior to temporal filtering between 0.009 and 0.2 Hz, and spatial filtering. A temporal average volume was used to create a multi-subject template guided by affine and nonlinear normalization to the Montreal Neurological Institute template. The Schaeffer 300 parcellation[76] was used to extract ROI time series. Adjacency matrices comprising Fisher-z transformed pairwise correlations ([300 * 299) / 2] = 44,850 edges) were set to 20% density prior to network calculations. These included the same measures described above. Weighted undirected networks were visualized in BrainNetViewer [77].

Network time series were generated and used in HMM-MAR inference as described above and as illustrated in Supplemental Figure 1. ROI time series were imported into MATLAB (190 time points by 300 ROIs) and a sliding window applied (30 TR, 1 TR step, for 90-second segments at 3 strides) to generate [timepoints – windows / step size + 1] 161 connectivity windows. Matrices were Fisher transformed and reorganized to 44,850 edges by 161 windows for HMM-MAR. HMM-MAR parameters were as described above. We conducted iterative inferencing of K from 3–7, selecting 3 based on the same criteria as described above. Dynamic features and graph theory network estimates for a 20% density threshold were derived as described above. As with mouse dynamic connectivity, we analyzed FO and dwell windows.

### Statistical analysis

Statistical analyses were conducted in MATLAB (MathWorks, Inc, Natick, MA) and GraphPad Prism (Boston, MA). We assumed heteroscedasticity, with all groups in both mouse and human portions of the work having an unequal number of subjects. In the specific case of the mouse imaging studies, we had unbalanced groups, with control mice having 3 age conditions (young, middle aged and aged) and the TG strains having only young and middle-aged conditions.

For static networks, we analyzed global metrics using a non-parametric Kruskal-Wallis analysis of variance (ANOVA) with group-wise post hoc tests using Dunn-Sidak’s correction. For node level analysis, we used permutation-based statistical comparisons as implemented in Permutation Analysis of Linear Models (PALM)[78]. In addition to reasonable handling of multiple nonparametric contrasts, PALM handles both corrections for multiple comparisons across ROI’s as well as multiple group-level contrast corrections. A linear model was constructed with the FSL GLM tool to inference age, Aβ and their interaction. Specifically, we tested: (1) main effect of age by contrasting aged vs middle-aged and young mice regardless of strain, (2) main effect of Aβ through contrasts between Aβ mice vs NTG mice of any age, and 3 additional contrasts designed to test age x Aβ interactions independently for 5XFAD and CRND8 mice. We hypothesized age-related reductions in connectivity and amyloid related increases in connectivity across sensorimotor and cognitive areas. Permutations were done over the entire dataset, with the following options: Fisher non-parametric contrasts with family-wise error correction for number of contrasts, two-tail distributions, data demeaning, accelerated tail estimation, with 10,000 total permutations (95! or approximately 1×10^148^ shuffles), and all resulting log-P values were false discovery rate (FDR) adjusted across ROIs. Contrast T statistics are reported here along with FDR corrected p values. For rejections of the null hypothesis per ROI, post hoc tests between group ROI values were then carried out using Dunn-Sidak tests.

Dynamic functional connectivity state features and graph measures per state were analyzed with linear mixed effects (LME) ANOVA (MATLAB *lme*). For mouse dynamic networks, we tested for the main effect of group (7 groups: NTG, TG, subdivided by age), main effect of state (5 states), and group x state interaction (either global or nodal level network measure ~ group*state + 1|subjects, where network measure is either global or nodal strength, efficiency or clustering coefficient). All resulting p-values for node-level comparisons were FDR adjusted to q ≤ 0.05 per term (main effect or interaction p values).

Human static and dynamic network comparisons, either at the global or nodal levels, followed the same statistical steps as the mouse networks, except for the following: (1) Mann-Whitney tests were used to compare low vs. high Aβ groups, (2) LME ANOVA compared 2 groups (either global or nodal level network measure ~ group*state + 1|subjects, where network measure is either global or nodal strength, efficiency or clustering coefficient), (3) node level comparisons were done for nodes within DMN areas, according to the Schaeffer 300 node parcellation for 17 networks[76].

## Results

### Amyloidosis alters functional network strength, transitivity, and global efficiency

**Fig 2.**
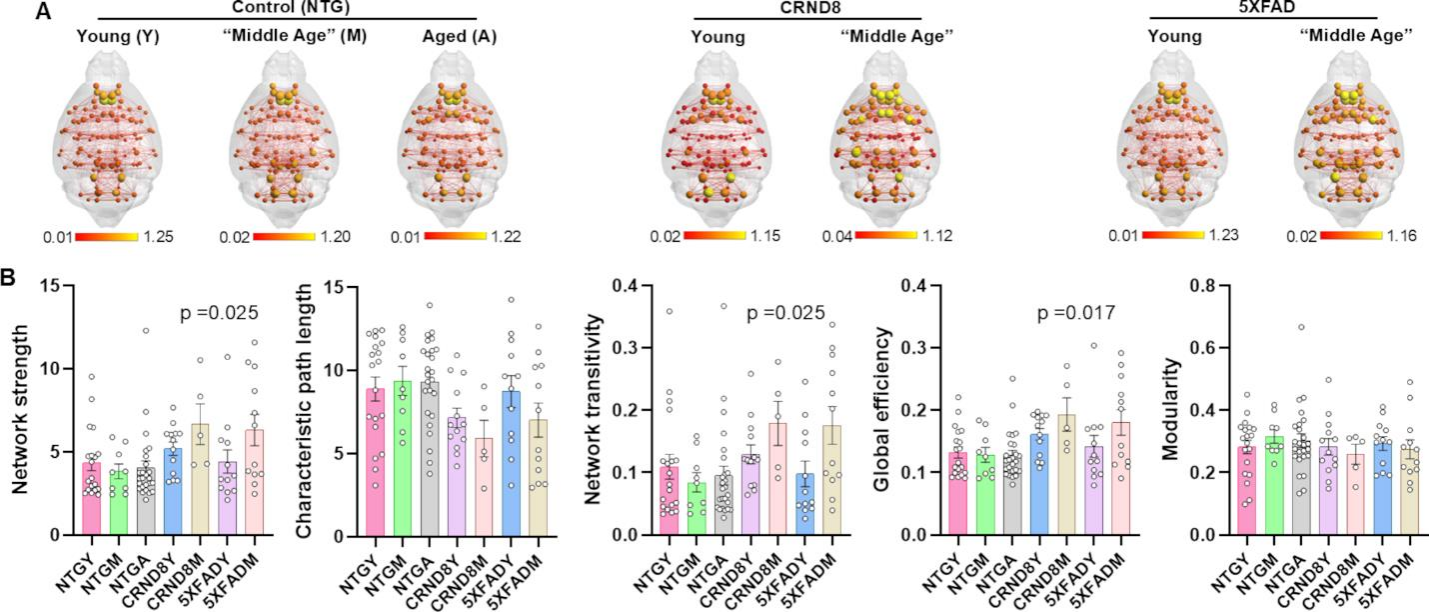
Amyloidosis, not age, alters global network strength, efficiency, and transitivity. A) Connectome maps of young (Y), middle age (M), and aged (A) non-transgenic (NTG) and transgenic (TG: TgCRND8, 5XFAD) mice. Maps visualized on mouse brain template[63] with node intensity indicating strength of connectivity and lines (edges) indicating Pearson correlation (edge weights). Scale bar indicates rescaled (0,1) edge weights. B) Global network measures. Data presented as mean ± standard error with overlaid scatter plots. Significant group differences, p<0.05 (Kruskal-Wallis test).

Network strength, clustering coefficient, and global efficiency significantly differed between the groups (Kruskal-Wallis ANOVA, p<0.05; Figure 2). Post hoc Dunn-Sidak tests did not reveal significant pairwise differences, although non-significant trends towards increased strength, clustering and efficiency were observed in TG strains compared to NTG (Figure 2).

**Fig 3.**
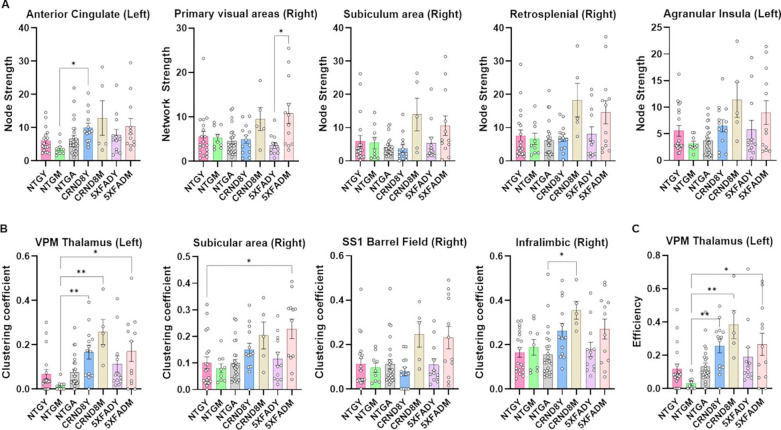
Amyloidosis alters node strength, clustering coefficient and efficiency across cognition and emotion related brain regions. Groups are as shown in Figure 2. A) Node strength. B) Clustering coefficient. C) Efficiency. All data presented as mean ± standard error with overlaid scatter plots. Significant differences tested across all nodes using linear mixed effects ANOVA (FDR corrected). Post hoc Dunn’s tests indicated by asterisks (*p<0.05, **p<0.01).

Nonparametric contrasts (FDR-adjusted) comparing node strength between young, middle aged and aged NTG and young and middle-aged TG groups showed a significant main effect of Aβ in anterior cingulate cortex (t_89_ = 17.8, p=0.045), agranular insular cortex (t_89_ = 17.7, p = 0.045), secondary motor cortex (t89 = 15.6, p = 0.049), superior colliculus-motor area (t_89_ = 17.9, p = 0.045) in the left hemisphere, and superior colliculus-motor area (t_89_ = 16.1, p = 0.049), inferior colliculus (t_89_ = 19.9, p = 0.042), floccular nodal region of the cerebellum (t_89_ = 16.0, p = 0.049) in the right hemisphere (Figure 3). An age x Aβ interaction was observed in the retrosplenial cortex (t_89_ = 15.9, p = 0.033), primary visual area (t_89_ = 18.6, p = 0.031), subicular region (t_89_ = 17.2, p = 0.031), and periaqueductal gray (t_89_ = 18.4, p = 0.031) in the right hemisphere. Main effect of age was observed in the right periaqueductal gray (t_89_ = 16.9, p = 0.045). No age x amyloid interactions for each Aβ strain independently were observed. Post hoc (Dunn-Sidak) pairwise contrasts are summarized in Figure 3A and Supplemental Figure 2A.

Nonparametric contrasts (FDR-adjusted) comparing clustering coefficients between groups showed a significant effect of Aβ in ventroposteromedial (VPM) (t_89_ = 29.9, p = 0.00069) and spinal nucleus of the trigeminal region (SpVr) (t_89_ = 19.6, p = 0.026) in left hemisphere and infralimbic cortex (t_89_ = 17.6, p=0.029) and subicular region (t_89_ = 19.6, p=0.029) in the right hemisphere. A significant age x Aβ interaction was observed in primary somatosensory area (t_89_ = 16.1, p = 0.046), and motor-related superior colliculus region (t_89_ = 16.1, p = 0.046) in the right hemisphere. No effect of age or age x amyloid interactions for each Aβ strain independently were observed. Post hoc (Dunn-Sidak) pairwise contrasts are summarized in Figure 3B and Supplemental Figure 2B.

Nonparametric contrasts (FDR-adjusted) comparing node efficiency between groups showed a significant effect of Aβ in VPM thalamus (t_89_ = 29.9, p = 0.001) in the left hemisphere. No significant age effect nor age related effect of Aβ was observed. No effect of age or age x amyloid interactions for grouped Aβ mice or for each Aβ strain independently were observed. Post hoc (Dunn-Sidak) pairwise contrasts are summarized in Figure 3C.

### Age and amyloidosis are associated with fluctuations in network strength, transitivity, and efficiency across functional connectome states

The average fractional occupancy (FO) per state across all groups was 0.20 ± 0.008 and the mean number of dwell windows per state was 41.8 ± 0.93.

**Fig 4.**
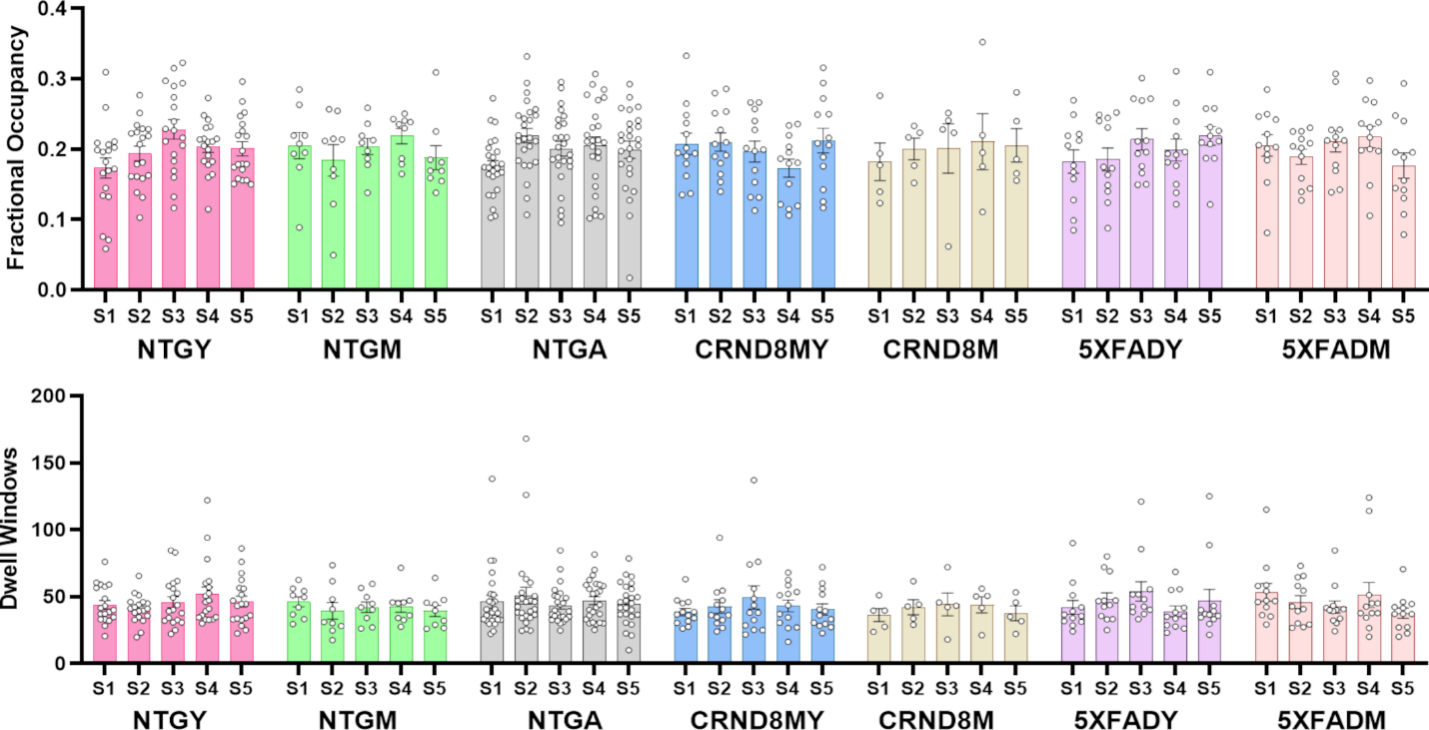
Stable dynamic functional network states in NTG and TG mice. Shown are fractional occupancy (fraction of total time spent in each state) and dwell windows (number of windows per each state). Groups are as shown in Figure 2. All data presented as mean ± standard error with overlaid scatter plots. Significant differences tested across all nodes using mixed effects ANOVA (FDR corrected). Post hoc Dunn’s tests indicated by asterisks (*p<0.05)

LME ANOVA revealed a significant state and state x group effect on state transition probabilities (state effect F_19,1900_ = 1.75, p = 0.02; state x group interaction F_114,1900_ = 1.35, p = 0.008). No differences in FO and dwell windows were observed (Figure 4). We did not observe differences in state switch rates.

**Fig 5.**
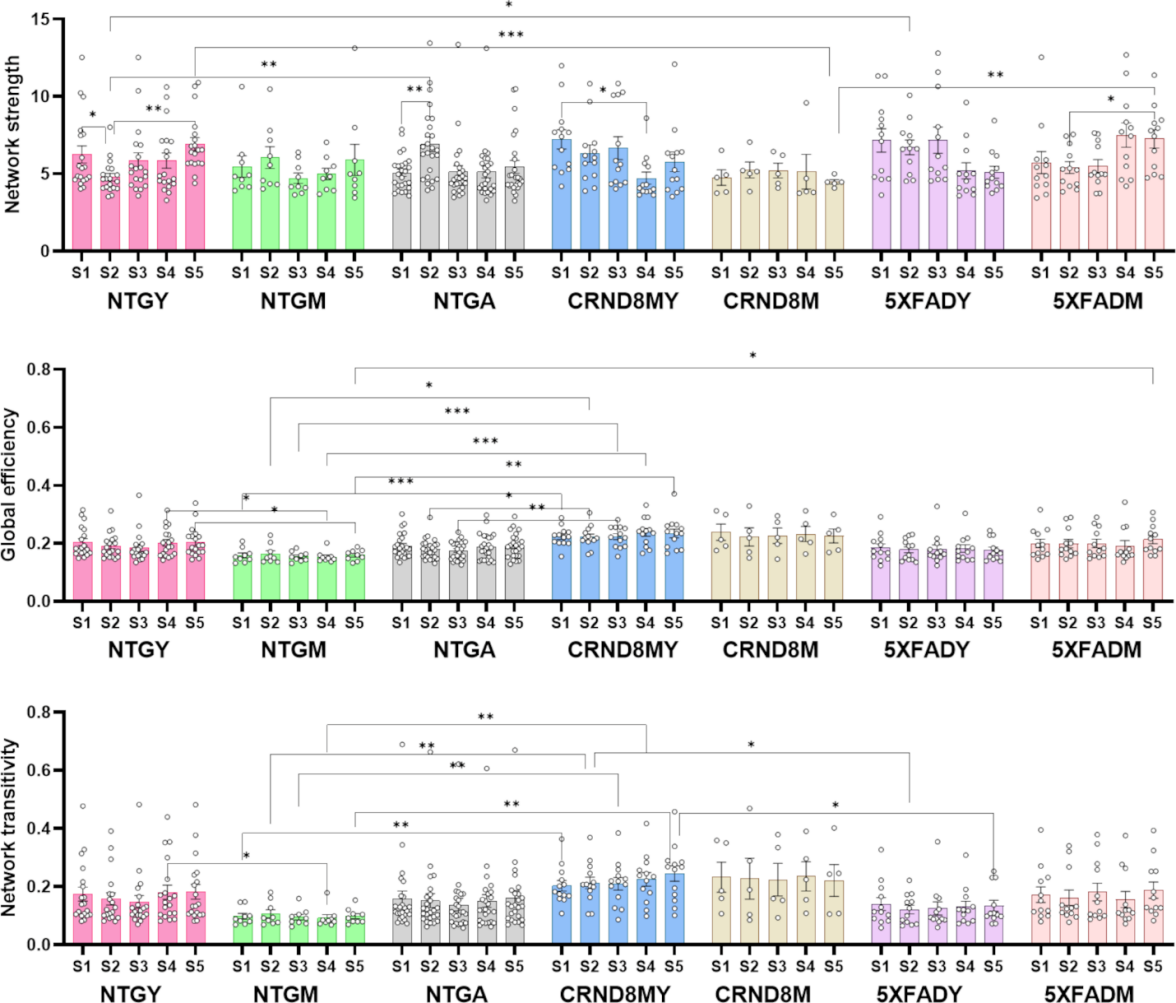
Amyloid associated group differences in strength, transitivity and global efficiency observed in static networks persist in dynamic functional networks. Groups are as shown in Figure 2. All data presented as mean ± standard error with overlaid scatter plots. Significant differences tested across all nodes using mixed effects ANOVA (FDR corrected). Post hoc Dunn’s tests indicated by asterisks (*p<0.05, **p<0.01, ***p<0.005).

A 2-way mixed effect (ME) ANOVA indicated a main effect of group (F_6, 88_ = 2.6, p = 0.025) and group x state interactions (F_22.5, 329.8_ = 2.6, p = 0.0001) on network strength. Pairwise comparisons using Tukey’s multiple comparison test show within group (state fluctuations, specific to Aβ groups) and between groups differences in network strength. These are summarized in Figure 5. Increased network strength in Aβ groups, observed in static networks, persisted across dynamic networks.

We observed main effects of state (F_3.7, 321.5_ = 2.5, p = 0.049) and group (F_6, 88_ = 3.9, p = 0.002) on global efficiency (2-way ME ANOVA; Figure 5). No significant group x state interactions were observed. Pairwise comparisons using Tukey’s multiple comparison test show within group (state fluctuations, specific to Aβ groups) and between groups differences in global efficiency. These are summarized in Figure 5.

We also observed main effects of state (F_3.7, 321.5_ = 2.5, p = 0.048) and group (F_6, 88_ = 2.3, p = 0.04) on network transitivity (2-way ME ANOVA; Figure 5). No significant group x state interactions were observed. Pairwise comparisons using Tukey’s multiple comparison test show within group (state fluctuations, specific to Aβ groups) and between groups differences in transitivity. These are summarized in Figure 5.

**Fig 6.**
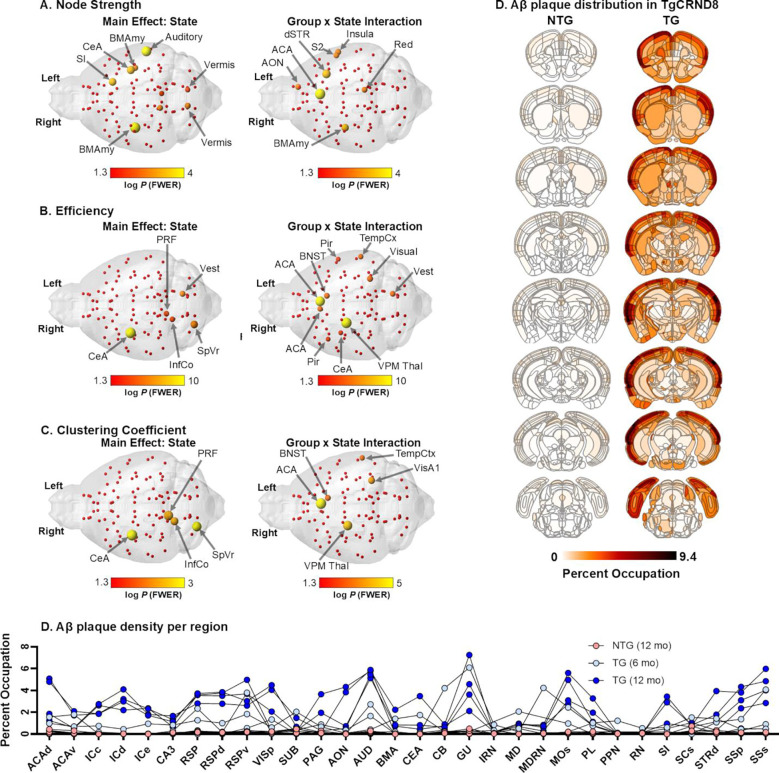
Group differences in node strength, efficiency and clustering coefficient vary across dynamic states in cognition and emotion related brain regions. A-C) Nodes showing main effect of state and group x state interactions for node strength (A), efficiency (B), and clustering coefficient (C). Scale bar color intensity indicates log p value. Significant differences tested across all nodes using linear mixed effects ANOVA (FDR corrected). D) Whole brain histology of Aβ plaque distribution in brains of an NTG and a TgCRND8 mouse. Scale bar indicates percent occupation of Aβ signal per ROI. E) Aβ plaque density per region. Data presented as individual data points per hemibrain (NTG, n=2; 6mo TG, n=3, 12mo TG, n=2). Abbreviations: ACA, anterior cingulate; IC, internal capsule; CA3, hippocampus; RSP, retrosplenium; VIS, visual cortex; PAG, periaqueductal grey; AON, anterior olfactory nucleus; AUD, auditory cortex; BMA, basomedial amygdala; BNST, bed nucleus stria terminalis; CEA, central amygdala; CB, cerebellum; GU, gustatory cortex; IRN, intermediate reticular nucleus; MD, mediodorsal thalamus; MDRN, midbrain reticular nucleus; MO, motor cortex; Pir, piriform; PL, prelimbic, RN, reticular nucleus; PPN, pedunculopontine nucleus; SI, substantia innominata; SC, superior colliculus; STR, striatum, SS, somatosensory; SpVr, spinal trigeminal nucleus region; VPM thal, ventroposteromedial thalamus; p, primary; s, secondary; d, dorsal; e, external; v, ventral.

LME ANOVA indicated a significant effect of state and a group x state interaction on node strength across several ROI nodes. The nodes are summarized in Figure 6A. The nodes with group x state interactions included left anterior cingulate (F_24,380_=3.3, FDR p = 0.00007), left anterior olfactory nucleus (AON) (F_24,380_=2.2, FDR p = 0.02), left dorsal striatum (F_24,380_=2.8, FDR p = 0.0008), left insula (F_24,380_=2.3, FDR p = 0.02), left supplemental somatosensory area (F_24,380_=2.1, FDR p = 0.02), left red nucleus (F_24,380_=2.2, FDR p = 0.02), and right basomedial amygdala (F_24,380_=2.6, FDR p = 0.003). The nodes with main effect of state included the left substantia innominata (F_4,380_=6.2, FDR p = 0.002), left central amygdala (F_4,380_=6.6, FDR p = 0.001), left auditory cortex (F_4,380_=8.3, FDR p = 0.001), left PAG (F_4,380_=3.9, FDR p = 0.00007), right superior colliculus (F_4,380_=4.5, FDR p = 0.02), left (F_4,380_=4.7, FDR p = 0.02) and right basomedial amygdala (F_4,380_=9.1, FDR p = 0.00005) and left (F_4,380_=3.9, FDR p = 0.04) and right cerebellar vermis (F_4,380_=4.8, FDR p = 0.02).

LME ANOVA indicated a significant effect of state and a group x state interaction on node clustering coefficient across several ROI nodes. The nodes are summarized in Figure 6C. The nodes with group x state interactions included left anterior cingulate (F_24,380_=3.5, FDR p = 0.00001), left primary visual area (F_24,380_=2.6, FDR p = 0.003), left temporal cortex (F_24,380_=2.2, FDR p = 0.03), left bed nucleus of stria terminalis (BNST) (F_24,380_=2.3, FDR p = 0.01), right VPM thalamus (F_24,380_=3.2, FDR p = 0.00008). Nodes with main effect of state included the right pontine reticular formation (F_4,380_=6.1, FDR p = 0.003), inferior colliculus (F_4,380_=5.1, FDR p = 0.01), right SpVr (F_4,380_=6.4, FDR p = 0.003) and right central amygdala (F_4,380_=7.3, FDR p = 0.001).

LME ANOVA indicated a significant effect of state and a group x state interaction on node efficiency across several ROI nodes. The nodes are summarized in Figure 6B. The nodes with group x state interactions included left (F_24,380_=3.4, FDR p = 0.00002) and right anterior cingulate (F_24,380_=2.2, FDR p = 0.02), left insula (F_24,380_=2.0, FDR p = 0.04), left temporal cortex (F_24,380_=2.2, FDR p = 0.02), left primary visual area (F_24,380_=2.5, FDR p = 0.004), left BNST (F_24,380_=2.3, FDR p = 0.009), left vestibular nucleus area (F_24,380_=2.3, FDR p = 0.009), right piriform cortex (F_24,380_=2.0, FDR p = 0.04), right central amygdala (F_24,380_=1.9, FDR p = 0.04), right VPM thalamus (F_24,380_=3.6, FDR p = 0.000006). Nodes with main effect of state included the right central amygdala (F_4,380_=7.3, FDR p = 0.001), right pontine reticular formation (F_4,380_=3.3, FDR p = 0.00007), vestibular nucleus area (F_4,380_=4.9, FDR p = 0.02), right inferior colliculus (F_4,380_=4.3, FDR p = 0.04), right SpVr (F_4,380_=6.2, FDR p = 0.004) and right central amygdala (F_4,380_=8.5, FDR p = 0.0001).

### Aβ plaques distribute across cortical and subcortical areas that overlap with functional network states affected by amyloidosis

Figure 6D–E illustrates semi-quantitative whole brain distribution of Aβ plaque signal in 6–12 m TgCRND8 mice. Plaque burden is measured as percent occupation of ROI. Highest densities of plaques are localized to outer layers of the cortical mantle, with notably greater percent Aβ occupation in anterior cingulate, frontal cortical areas, somatosensory, motor, and auditory regions.

### Aβ plaque associated increases in DMN connectome strength and state fluctuations

**Fig 7.**
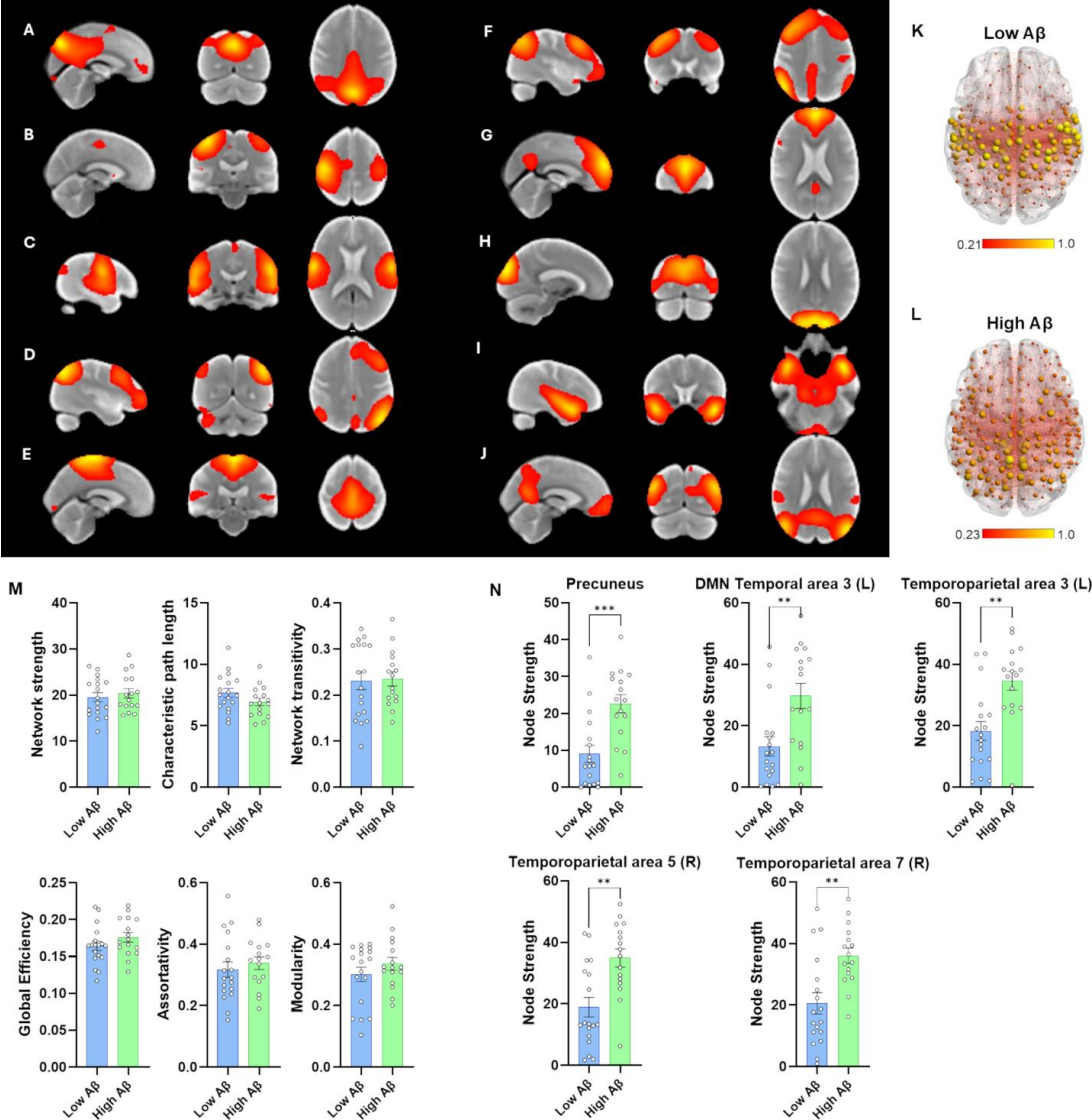
High Aβ plaque density is linked to greater node strength in default mode network areas. A) Default mode network areas. B) Right sensorimotor areas. C) Auditory areas. D) Left frontoparietal areas. E) Sensorimotor areas. F) Right frontoparietal areas. G) Executive areas. H) Medial visual areas. I) Auditory areas. J) Lateral visual areas. K) Group averaged functional connectome of low Aβ. L) Group averaged functional connectome of high Aβ. M) Global network measures. N) Connectivity strength in nodes of the default mode network. All data presented as mean ± standard error with overlaid scatter plots. Significant differences tested across all nodes using mixed effects ANOVA (FDR corrected). Post hoc Dunn’s tests indicated by asterisks (**p<0.01, ***p<0.005).

Group ICA revealed several well-established functional connectivity networks (Figure 7A–J). Medial and lateral visual cortical areas, auditory, sensorimotor, visuospatial systems, executive control, and dorsal visual stream were observed, as previously reported[79].

Group averaged functional connectomes showing nodal strength organization in brains of low and high Aβ subjects are shown in Figure 7K–L. No significant differences in global network metrics were observed between low and high Aβ groups (Figure 7M). One-way repeated measures ME ANOVA indicated a group x ROI interaction within the DMN (F_7.8,251.3_ = 3.0, p = 0.003). Post hoc analysis of nodes within the DMN indicated significantly greater node strength in precuneus (p=0.0003, q=0.02), temporal area of the DMN (p=0.004, q=0.046) and temporoparietal areas 3 (p=0.0007, q=0.02), 5 (p=0.0009, q=0.02) and 7 (p=0.001, q=0.02) of high compared to low Aβ (Figure 7N).

**Fig 8.**
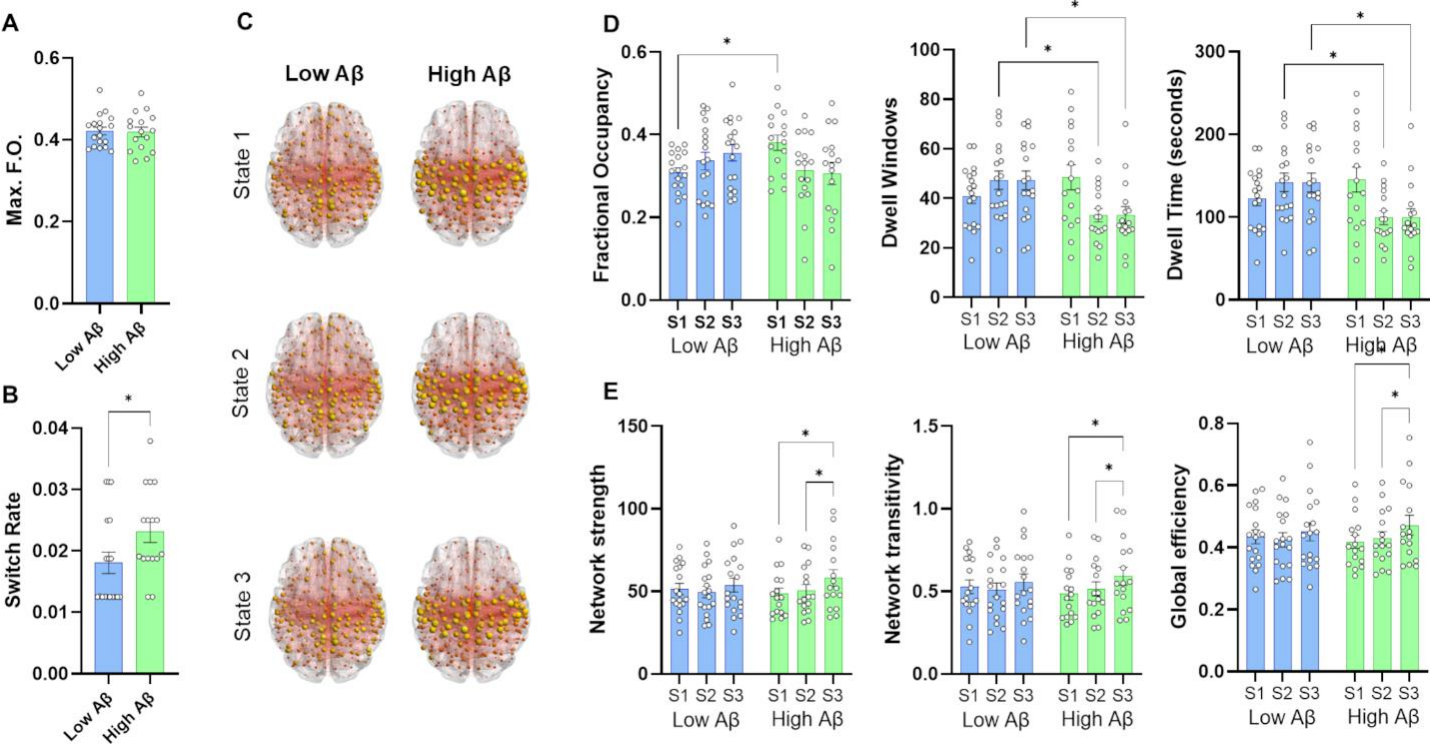
High amyloid burden is associated with high rates of state switching and instability of network strength, transitivity, and global efficiency across states. A) Maximum fractional occupancy per subject. B) State switch rates. C) Group-averaged dynamic functional connectome maps across 3 states for low and high Aβ. E) Network strength, transitivity, and global efficiency. All data presented as mean ± standard error with overlaid scatter plots. Significant differences tested across all nodes using mixed effects ANOVA (FDR corrected). Post hoc Dunn’s tests indicated by asterisks (*p<0.05).

Mean FO across subjects was 0.33 ± 0.009 and mean dwell windows, 40.6 ± 1.1, supporting stable state durations. While maximum FO did not differ between groups, high Aβ subjects had greater switch rates between than low Aβ subjects (Mann-Whitney test p = 0.03) (Figure 8A–B). Thus, Aβ subjects had faster rates of switching between states. In contrast to the effect of Aβ on transition probabilities in mice, no differences in state transition probabilities were observed between low and high Aβ. Figure 8C shows mean functional connectome maps for low and high Aβ groups. As observed with static connectomes, a greater node strength is observed in high Aβ relative to low in posterior medial and lateral temporal and parietal cortical nodes within broader DMN areas. A group x state interaction was observed for FO, with greater state 1 FO in high vs low Aβ (2-way ME ANOVA; F_1.68,80.7_ = 5, p = 0.013; post hoc test p = 0.0028 (FDR adjusted), state 1 low vs high Aβ). A group x state interaction was observed for dwell windows, with lower values observed in high vs low Aβ (dwell windows: F_1.8,58.6_ = 6.7, p = 0.003; state 1 and 2) (Figure 8D).

A 2-way ME ANOVA indicated a main effect of state on node network strength, with high Aβ subjects having higher network strength in state 3 vs state 1 and 2 (F_1.8,60_=9.0, p=0.0005; Tukey’s post hoc test p = 0.01 state 3 vs 1, p = 0.03 state 3 vs 2). A similar effect of high Aβ was observed on network transitivity and global efficiency (transitivity: F_1.8,60_=9.2, p=0.0004; Tukey’s post hoc test p = 0.006 state 3 vs 1, p = 0.04 state 3 vs 2; efficiency: F_1.8,60_=10.2, p=0.0006; Tukey’s post hoc test p = 0.01 state 3 vs 1, p = 0.03 state 3 vs 2) (Figure 8B). LME ANOVA indicated node level differences between low and high Aβ that did not survive FDR correction.

## Discussion

Our results indicate that Aβ is associated with increased static functional connectivity and network integration in both mice and humans but has dissociable effects on dynamic network organization across species. Specifically, dynamic network topology remained stable across states in Aβ mice, whereas human subjects with high Aβ exhibited increased state switching and altered state-dependent network measures. These results suggest that Aβ alone is insufficient to reproduce the dynamic network instability observed in human preclinical Alzheimer’s disease.

The present data agree with previous studies reporting increased functional connectivity between DMN-like areas in transgenic AβPP-bearing AD mice[19, 80]. We observed differences across TG and NTG groups, with the former having on average high connectivity strength, global efficiency and transitivity. At the nodal level, differences in these network measures were observed in anterior cingulate, retrosplenial cortex, subiculum, and primary visual areas, which are regions critical to cognitive and sensory processing, and that are known to accumulate Aβ plaques. Shah et al[19] were among the first to report hypersynchronous BOLD in both Tg2576 and PDAPP mice in areas that were also found here to have high node strength in middle aged TgCRND8 and 5XFAD mice. This was observed during pre-plaque stages in Tg2576 and PDAPP mice, both strains having single AD mutations. At later stages, functional connectivity declined significantly in their study[19], whereas in our study we observed elevated node strength in several regions in young TgCRND8 mice that already contain plaque deposits as early as 3mo and dense core plaques by 5mo [53]. Interestingly, node strength was mostly increased in middle age 5XFAD mice (15–19mo), which have elevated brain Aβ plaques and impaired contextual fear memory at 14mo[54]. The elevated connectivity strength persisted across more areas in both TG strains during 15–19mo. This is an important distinction between the studies since all strains produce human mutant Aβ and develop plaques, albeit at different ages and at different rates. TgCRND8 mice have double mutations while the diversity-variant 5XFAD we used here is on a mixed F1 B6D2 strain that harbors five AD mutations. An important distinction is thus that the PDAβPP and Tg2576 have single mutations, and in the case of the latter strain, these have vascular Aβ (as does the TgCRND8) and develop plaques more slowly. Still, this distinction does not explain why in our study we observed only elevated node strength, even at the later age group of TG mice. The discrepancy may be due to the analysis approach, with Shah et al[19] analyzing single pairwise functional connectivity which differs from our calculation of node strength. The approach used by Shah et al[19] is a specific readout for pairwise changes between two nodes over time, whereas node strength can remain elevated for one node if its pairwise connectivity is strong with any other set of nodes in a network. One is physiologically grounded and the other based on a statistical measure of node ‘prominence’ within a network (strength of overall ‘ties’ within a network). Work by Grandjean and colleagues[81] evaluated the effects of vascular, parenchymal, and intracellular Aβ in 3 strains of amyloid mice that preferentially deposit plaques in these compartments[81]. Within and between ICA component analysis was carried out, which showed reduced within-component functional connectivity (including cingulate-ventral hippocampus) and increased between-component connectivity (including prefrontal-cingulate). This finding supports the notion of reorganization of functional connectivity across groups of nodes, perhaps as part of the compensatory response to Aβ accumulation. It also should be noted that additional work by Shah et al[80] correlated increased pre-plaque functional connectivity with spatial learning deficits, based on performance on a Morris Water Maze task in the AβPP^NL-F^ knock-in mouse (Swedish/Iberian double mutations)[80]. A limitation of the present work is that we did not include behavioral assays for cognitive performance to evaluate connectome measures as a function of cognition behaviors.

The main objective of this study was to evaluate how Aβ alters dynamic functional connectivity. Specifically, we were interested in evaluating network-based measures of connectivity strength, efficiency and clustering coefficient, and other measures of network topology that may shed light on the effects of Aβ, both globally and locally across nodes. To our knowledge, there have been no studies to date that have examined how Aβ affects HMM-derived fMRI connectivity network states, a question that can be feasibly studied in mice engineered to express Aβ. There have, however, been studies that have identified hypersynchronous activity of single cell recordings[82] and increases in spontaneous synaptic activity in hippocampus of APP-PS1 mice[83]. It is these consistently reported mechanisms that might produce static and dynamic network changes that vary as a function of specific AD pathologies and their combinations. BOLD fMRI co-activations over large regions of the cortex may be linked to shifts in hypersynchronous activity across specific behaviorally or cognitively relevant states; although, the specific subsets of neurons, their physiological and biochemical properties, and how their elevated cross-population synchronicity modify broader networks in specific ways that lead to altered strength, efficiency and clustering over states, remain unknown at this time. One possible contribution could be from changes in quasi-periodic patterns (QPPs) that are known to contribute to spatial and temporal dynamics in resting state fMRI[84]. Middle aged (18mo) Tg2576 mice that develop Aβ plaques around 9–11 mo had lower occurrence rates for QPPs compared to age-matched controls[85]. DMN-like areas also showed reduced anticorrelated functional connectivity with task-positive areas of the mouse brain[85]. Our results suggest that the effect of Aβ on static network measures is dissociable from the dynamic functional network stability that was observed in mice. The relatively stable state patterns may be due to lack of behavior or the effects of sedation, which is not the case of human functional connectomes. However, unknown pathologies present in human connectomes, and not in our Aβ mice, may also explain our results. Combining AD relevant pathologies may bring mouse dynamic connectomes closer to representing the state dependent variations observed in human subjects.

Previous work using K means clustering determined that fMRI timeseries in mice could be segmented into 6 states (co-activation patterns) with similar network features[68]. Such an approach captures rodent network features that may be conserved and translatable to primate networks[51]. Twenty distinct explanatory vectors were identified using a dictionary learning algorithm, which also identified relationships of several of these states (atoms) to chronic stress exposure[70]. This approach in mice may model aspects of human studies identifying links between dynamic functional connectivity and neuropsychiatry condition-specific network states[39]. The HMM variant used here applied a mixture of Gaussians under variational Bayes framework to find a lower evidence bound solution to segmenting network-based time series into predefined states across all subjects. Thus, while it was found that the networks could be segmented into 5 states but not 6 or greater, this was determined across the entire cohort of mice that varied by age and Aβ conditions (and by sex). Thus, a more uniform cohort of mice may may produce a different outcome (how many states are segmented). Further, the inclusion of behavioral assessments[86], especially close enough in time to the imaging as in previous work in rats[87], may raise the predictive validity of these approaches to distinct behavioral states[88]. It should also be noted, however, that a significant limitation to this is end is that the segmentation of functional MRI based network timeseries is affected by the use of either sedatives or gaseous anesthetics[67, 68, 89], but it is also likely impacted by excess movements in rodents imaged awake[90].

Human functional connectome results are consistent with previous reports. In terms of static network strength, we observed increased node strength in precuneus, and other regions of the DMN. Previous research indicated that Aβ (and tau) in cognitively unimpaired subjects (~60 years of age) is associated with increased functional connectivity between precuneus and anterior hippocampus[9]. This result is consistent with in the observed increases in strength, efficiency and transitivity in DMN-like areas of Aβ mouse static networks. As in mouse networks, the effect of Aβ plaques may include neuronal, synaptic, and vascular deficits that can explain the present results. Pathological changes in response to Aβ could provide the basis for within-state changes in network measures, where some states are visited more often than others. A reduction in frequency of visits to one of three identified states was observe in AD (females but not males)[49]. This differs from our results, which included mostly women, and were pre-selected for not having signs of cognitive impairment. Visits to specific states vary as a function of dementia risk factors and self-reports of cognitive performance[43]. Interestingly, frequent visits to a single state are observed in small vessel disease[91, 92] and appear to distinguish small vessel disease from AD[93]. This suggests a role for either changes in small vessel structure and function or a role of astrocytes that regulate neurovascular coupling and local perfusion.

There are several limitations in our study that should be noted. First, as mentioned above, the use of anesthetic and sedative are sure to impact what aspects of network connectivity are captured in mice. The protocol used here, however, is often used across laboratories and offers a good solution to image sedated mice and comparing results across mouse imaging laboratories. Awake mouse studies are feasible. However, the use of head restraint over relatively long scanning sessions and the interference on image quality produced by movements of the body would also raise marked concerns. The use of simple behavioral tasks to image well-trained awake mice may be a good solution and may facilitate interpretations when analyzing dynamic functional connectivity networks. Recent work using a simple motor task along with the use of the movement-insensitive zero TE sequence seems like a strong future direction[94]. Implementation of this approach, or similar methods, across preclinical laboratories should improve harmonization of data collected in awake mice. Also, inclusion of behavioral and molecular ‘explanatory’ variables[60] will raise the extent to which the future data provide an in-depth understanding of Aβ effects on dynamic networks inferenced with HMMs. Another limitation worth noting is that our human scans were not only in a limited cohort but were selected for specific *a priori* features. This biases our results in the context of the effect of Aβ on functional connectomes. However, this was important to the goal of evaluating the effect of Aβ in our study. Future studies should include a variety of phenotypes and pathologies to ensure statistical robustness in capturing the effects Aβ relative to other factors.

## Supplementary Material

Supplement 1

## References

[1] Masters CL, Bateman R, Blennow K, Rowe CC, Sperling RA, Cummings JL. Alzheimer’s disease. Nat Rev Dis Primers. 2015;1:15056. Epub 20151015. doi: 10.1038/nrdp.2015.56. 27188934

[2] Herrup K. The case for rejecting the amyloid cascade hypothesis. Nat Neurosci. 2015;18(6):794–9. doi: 10.1038/nn.4017. 26007212

[3] Zhang X, Zeng Q, Wang Y, Jin Y, Qiu T, Li K, Alteration of functional connectivity network in population of objectively-defined subtle cognitive decline. Brain Commun. 2024;6(1):fcae033. Epub 20240209. doi: 10.1093/braincomms/fcae033. 38425749 PMC10903975

[4] Buckner RL, Sepulcre J, Talukdar T, Krienen FM, Liu H, Hedden T, Cortical hubs revealed by intrinsic functional connectivity: mapping, assessment of stability, and relation to Alzheimer’s disease. J Neurosci. 2009;29(6):1860–73. doi: 10.1523/JNEUROSCI.5062-08.2009. 19211893 PMC2750039

[5] Buckner RL, Snyder AZ, Shannon BJ, LaRossa G, Sachs R, Fotenos AF, Molecular, structural, and functional characterization of Alzheimer’s disease: evidence for a relationship between default activity, amyloid, and memory. J Neurosci. 2005;25(34):7709–17. doi: 10.1523/JNEUROSCI.2177-05.2005. 16120771 PMC6725245

[6] Hedden T, Van Dijk KR, Becker JA, Mehta A, Sperling RA, Johnson KA, Disruption of functional connectivity in clinically normal older adults harboring amyloid burden. J Neurosci. 2009;29(40):12686–94. doi: 10.1523/JNEUROSCI.3189-09.2009. 19812343 PMC2808119

[7] Wang J, Zuo X, Dai Z, Xia M, Zhao Z, Zhao X, Disrupted functional brain connectome in individuals at risk for Alzheimer’s disease. Biol Psychiatry. 2013;73(5):472–81. Epub 20120425. doi: 10.1016/j.biopsych.2012.03.026. 22537793

[8] Pini L, Brusini L, Griffa A, Cruciani F, Allali G, Frisoni GB, Functional dynamic network connectivity differentiates biological patterns in the Alzheimer’s disease continuum. Neurobiol Dis. 2025;208:106866. Epub 20250311. doi: 10.1016/j.nbd.2025.106866. 40081429

[9] Fischer L, Adams JN, Molloy EN, Vockert N, Tremblay-Mercier J, Remz J, Differential effects of aging, Alzheimer’s pathology, and APOE4 on longitudinal functional connectivity and episodic memory in older adults. Alzheimers Res Ther. 2025;17(1):91. Epub 20250425. doi: 10.1186/s13195-025-01742-6. 40281595 PMC12023467

[10] Pereira JB, Strandberg TO, Palmqvist S, Volpe G, van Westen D, Westman E, Amyloid Network Topology Characterizes the Progression of Alzheimer’s Disease During the Predementia Stages. Cereb Cortex. 2018;28(1):340–9. doi: 10.1093/cercor/bhx294. 29136123 PMC6454565

[11] Myers N, Pasquini L, Gottler J, Grimmer T, Koch K, Ortner M, Within-patient correspondence of amyloid-beta and intrinsic network connectivity in Alzheimer’s disease. Brain. 2014;137(Pt 7):2052–64. Epub 20140426. doi: 10.1093/brain/awu103. 24771519 PMC4065018

[12] Dai Z, He Y. Disrupted structural and functional brain connectomes in mild cognitive impairment and Alzheimer’s disease. Neurosci Bull. 2014;30(2):217–32. Epub 20140415. doi: 10.1007/s12264-013-1421-0. 24733652 PMC5562665

[13] Dai Z, Yan C, Li K, Wang Z, Wang J, Cao M, Identifying and Mapping Connectivity Patterns of Brain Network Hubs in Alzheimer’s Disease. Cereb Cortex. 2015;25(10):3723–42. Epub 20141019. doi: 10.1093/cercor/bhu246. 25331602

[14] Susanto TA, Pua EP, Zhou J, Alzheimer’s Disease Neuroimaging I. Cognition, brain atrophy, and cerebrospinal fluid biomarkers changes from preclinical to dementia stage of Alzheimer’s disease and the influence of apolipoprotein e. J Alzheimers Dis. 2015;45(1):253–68. doi: 10.3233/JAD-142451. 25524955

[15] Brier MR, Thomas JB, Ances BM. Network dysfunction in Alzheimer’s disease: refining the disconnection hypothesis. Brain Connect. 2014;4(5):299–311. doi: 10.1089/brain.2014.0236. 24796856 PMC4064730

[16] Canu E, Agosta F, Mandic-Stojmenovic G, Stojkovic T, Stefanova E, Inuggi A, Multiparametric MRI to distinguish early onset Alzheimer’s disease and behavioural variant of frontotemporal dementia. Neuroimage Clin. 2017;15:428–38. Epub 20170525. doi: 10.1016/j.nicl.2017.05.018. 28616383 PMC5458769

[17] Cieri F, Zhuang X, Cordes D, Kaplan N, Cummings J, Caldwell J, Relationship of sex differences in cortical thickness and memory among cognitively healthy subjects and individuals with mild cognitive impairment and Alzheimer disease. Alzheimers Res Ther. 2022;14(1):36. Epub 20220222. doi: 10.1186/s13195-022-00973-1. 35193682 PMC8864917

[18] Jones DT, Graff-Radford J, Lowe VJ, Wiste HJ, Gunter JL, Senjem ML, Tau, amyloid, and cascading network failure across the Alzheimer’s disease spectrum. Cortex. 2017;97:143–59. Epub 20171003. doi: 10.1016/j.cortex.2017.09.018. 29102243 PMC5773067

[19] Shah D, Praet J, Latif Hernandez A, Hofling C, Anckaerts C, Bard F, Early pathologic amyloid induces hypersynchrony of BOLD resting-state networks in transgenic mice and provides an early therapeutic window before amyloid plaque deposition. Alzheimers Dement. 2016;12(9):964–76. Epub 20160421. doi: 10.1016/j.jalz.2016.03.010. 27107518

[20] Latif-Hernandez A, Shah D, Craessaerts K, Saido T, Saito T, De Strooper B, Subtle behavioral changes and increased prefrontal-hippocampal network synchronicity in APP(NL-G-F) mice before prominent plaque deposition. Behav Brain Res. 2019;364:431–41. Epub 20171120. doi: 10.1016/j.bbr.2017.11.017. 29158112

[21] Grandjean J, Schroeter A, He P, Tanadini M, Keist R, Krstic D, Early alterations in functional connectivity and white matter structure in a transgenic mouse model of cerebral amyloidosis. J Neurosci. 2014;34(41):13780–9. doi: 10.1523/JNEUROSCI.4762-13.2014. 25297104 PMC6608375

[22] Shah D, Jonckers E, Praet J, Vanhoutte G, Delgado YPR, Bigot C, Resting state FMRI reveals diminished functional connectivity in a mouse model of amyloidosis. PLoS One. 2013;8(12):e84241. Epub 2013/12/21. doi: 10.1371/journal.pone.0084241. 24358348 PMC3866274

[23] Manno FAM, Isla AG, Manno SHC, Ahmed I, Cheng SH, Barrios FA, Early Stage Alterations in White Matter and Decreased Functional Interhemispheric Hippocampal Connectivity in the 3xTg Mouse Model of Alzheimer’s Disease. Front Aging Neurosci. 2019;11:39. Epub 20190322. doi: 10.3389/fnagi.2019.00039. 30967770 PMC6440287

[24] Ben-Nejma IRH, Keliris AJ, Daans J, Ponsaerts P, Verhoye M, Van der Linden A, Increased soluble amyloid-beta causes early aberrant brain network hypersynchronisation in a mature-onset mouse model of amyloidosis. Acta Neuropathol Commun. 2019;7(1):180. Epub 20191114. doi: 10.1186/s40478-019-0810-7. 31727182 PMC6857138

[25] Parent MJ, Zimmer ER, Shin M, Kang MS, Fonov VS, Mathieu A, Multimodal Imaging in Rat Model Recapitulates Alzheimer’s Disease Biomarkers Abnormalities. J Neurosci. 2017;37(50):12263–71. Epub 20171102. doi: 10.1523/JNEUROSCI.1346-17.2017. 29097597 PMC5729194

[26] Behfar Q, Behfar SK, von Reutern B, Richter N, Dronse J, Fassbender R, Graph Theory Analysis Reveals Resting-State Compensatory Mechanisms in Healthy Aging and Prodromal Alzheimer’s Disease. Front Aging Neurosci. 2020;12:576627. Epub 20201022. doi: 10.3389/fnagi.2020.576627. 33192468 PMC7642892

[27] Cha WJ, Chumin EJ, Yi D, Byun MS, Jung JH, Ahn H, Amyloid-related default mode network hyperconnectivity and longitudinal decline in network distinctiveness in preclinical Alzheimer’s disease. Alzheimers Dement. 2026;22(2):e71025. doi: 10.1002/alz.71025. 41612924 PMC12856376

[28] Toussaint PJ, Maiz S, Coynel D, Doyon J, Messe A, de Souza LC, Characteristics of the default mode functional connectivity in normal ageing and Alzheimer’s disease using resting state fMRI with a combined approach of entropy-based and graph theoretical measurements. Neuroimage. 2014;101:778–86. Epub 20140809. doi: 10.1016/j.neuroimage.2014.08.003. 25111470

[29] Faskhodi MM, Einalou Z, Dadgostar M. Diagnosis of Alzheimer’s disease using resting-state fMRI and graph theory. Technol Health Care. 2018;26(6):921–31. doi: 10.3233/THC-181312. 30124458

[30] Liu Y, Yu C, Zhang X, Liu J, Duan Y, Alexander-Bloch AF, Impaired long distance functional connectivity and weighted network architecture in Alzheimer’s disease. Cereb Cortex. 2014;24(6):1422–35. Epub 20130111. doi: 10.1093/cercor/bhs410. 23314940 PMC4215108

[31] Mijalkov M, Vereb D, Canal-Garcia A, Hinault T, Volpe G, Pereira JB, Nonlinear changes in delayed functional network topology in Alzheimer’s disease: relationship with amyloid and tau pathology. Alzheimers Res Ther. 2023;15(1):112. Epub 20230616. doi: 10.1186/s13195-023-01252-3. 37328909 PMC10273754

[32] Sanz-Arigita EJ, Schoonheim MM, Damoiseaux JS, Rombouts SA, Maris E, Barkhof F, Loss of ‘small-world’ networks in Alzheimer’s disease: graph analysis of FMRI resting-state functional connectivity. PLoS One. 2010;5(11):e13788. Epub 20101101. doi: 10.1371/journal.pone.0013788. 21072180 PMC2967467

[33] Contreras JA, Avena-Koenigsberger A, Risacher SL, West JD, Tallman E, McDonald BC, Resting state network modularity along the prodromal late onset Alzheimer’s disease continuum. Neuroimage Clin. 2019;22:101687. Epub 20190122. doi: 10.1016/j.nicl.2019.101687. 30710872 PMC6357852

[34] Daianu M, Dennis EL, Jahanshad N, Nir TM, Toga AW, Jack CR Jr., Alzheimer’s Disease Disrupts Rich Club Organization in Brain Connectivity Networks. Proc IEEE Int Symp Biomed Imaging. 2013:266–9. doi: 10.1109/ISBI.2013.6556463. 24953139 PMC4063983

[35] Lorenzini L, Ingala S, Collij LE, Wottschel V, Haller S, Blennow K, Eigenvector centrality dynamics are related to Alzheimer’s disease pathological changes in non-demented individuals. Brain Commun. 2023;5(3):fcad088. Epub 20230328. doi: 10.1093/braincomms/fcad088. 37151225 PMC10156145

[36] Munoz-Moreno E, Tudela R, Lopez-Gil X, Soria G. Early brain connectivity alterations and cognitive impairment in a rat model of Alzheimer’s disease. Alzheimers Res Ther. 2018;10(1):16. Epub 20180207. doi: 10.1186/s13195-018-0346-2. 29415770 PMC5803915

[37] Kesler SR, Acton P, Rao V, Ray WJ. Functional and structural connectome properties in the 5XFAD transgenic mouse model of Alzheimer’s disease. Netw Neurosci. 2018;2(2):241–58. Epub 20180601. doi: 10.1162/netn_a_00048. 30215035 PMC6130552

[38] Simon ZD, McFarland KN, Golde TE, Chakrabarty P, Febo M. Sex-dependent effect of amyloidosis on functional network ‘hub’ topology is associated with downregulated neuronal gene signatures in the APPswe/PSEN1dE9 double transgenic mouse. J Alzheimers Dis. 2025;108(2):893–913. Epub 20250917. doi: 10.1177/13872877251378778. 40961168

[39] Rashid B, Damaraju E, Pearlson GD, Calhoun VD. Dynamic connectivity states estimated from resting fMRI Identify differences among Schizophrenia, bipolar disorder, and healthy control subjects. Front Hum Neurosci. 2014;8:897. Epub 20141107. doi: 10.3389/fnhum.2014.00897. 25426048 PMC4224100

[40] Lin SJ, Kolind S, Liu A, McMullen K, Vavasour I, Wang ZJ, Both Stationary and Dynamic Functional Interhemispheric Connectivity Are Strongly Associated With Performance on Cognitive Tests in Multiple Sclerosis. Front Neurol. 2020;11:407. Epub 20200604. doi: 10.3389/fneur.2020.00407. 32581993 PMC7287147

[41] Qian S, Yang Q, Cai C, Dong J, Cai S. Spatial-Temporal Characteristics of Brain Activity in Autism Spectrum Disorder Based on Hidden Markov Model and Dynamic Graph Theory: A Resting-State fMRI Study. Brain Sci. 2024;14(5). Epub 20240517. doi: 10.3390/brainsci14050507. PMC11118919 38790485

[42] Schumacher J, Peraza LR, Firbank M, Thomas AJ, Kaiser M, Gallagher P, Dynamic functional connectivity changes in dementia with Lewy bodies and Alzheimer’s disease. Neuroimage Clin. 2019;22:101812. Epub 20190403. doi: 10.1016/j.nicl.2019.101812. 30991620 PMC6462776

[43] Dautricourt S, Gonneaud J, Landeau B, Calhoun VD, de Flores R, Poisnel G, Dynamic functional connectivity patterns associated with dementia risk. Alzheimers Res Ther. 2022;14(1):72. Epub 20220523. doi: 10.1186/s13195-022-01006-7. 35606867 PMC9128270

[44] Calhoun VD, Miller R, Pearlson G, Adali T. The chronnectome: time-varying connectivity networks as the next frontier in fMRI data discovery. Neuron. 2014;84(2):262–74. Epub 20141022. doi: 10.1016/j.neuron.2014.10.015. 25374354 PMC4372723

[45] Zalesky A, Fornito A, Cocchi L, Gollo LL, Breakspear M. Time-resolved resting-state brain networks. Proc Natl Acad Sci U S A. 2014;111(28):10341–6. Epub 20140630. doi: 10.1073/pnas.1400181111. 24982140 PMC4104861

[46] Bassett DS, Wymbs NF, Porter MA, Mucha PJ, Carlson JM, Grafton ST. Dynamic reconfiguration of human brain networks during learning. Proc Natl Acad Sci U S A. 2011;108(18):7641–6. Epub 20110418. doi: 10.1073/pnas.1018985108. 21502525 PMC3088578

[47] Nguyen TT, Kovacevic S, Dev SI, Lu K, Liu TT, Eyler LT. Dynamic functional connectivity in bipolar disorder is associated with executive function and processing speed: A preliminary study. Neuropsychology. 2017;31(1):73–83. Epub 20161024. doi: 10.1037/neu0000317. 27775400 PMC5471616

[48] Sendi MSE, Zendehrouh E, Fu Z, Liu J, Du Y, Mormino E, Disrupted Dynamic Functional Network Connectivity Among Cognitive Control Networks in the Progression of Alzheimer’s Disease. Brain Connect. 2023;13(6):334–43. Epub 20210907. doi: 10.1089/brain.2020.0847. 34102870 PMC10442683

[49] Sendi MSE, Zendehrouh E, Ellis CA, Fu Z, Chen J, Miller RL, The link between static and dynamic brain functional network connectivity and genetic risk of Alzheimer’s disease. Neuroimage Clin. 2023;37:103363. Epub 20230227. doi: 10.1016/j.nicl.2023.103363. 36871405 PMC9999198

[50] Wang Q, Chen B, Zhong X, Hou L, Zhang M, Yang M, Static and dynamic functional connectivity variability of the anterior-posterior hippocampus with subjective cognitive decline. Alzheimers Res Ther. 2022;14(1):122. Epub 20220903. doi: 10.1186/s13195-022-01066-9. 36057586 PMC9440588

[51] Gutierrez-Barragan D, Ramirez JSB, Panzeri S, Xu T, Gozzi A. Evolutionarily conserved fMRI network dynamics in the mouse, macaque, and human brain. Nat Commun. 2024;15(1):8518. Epub 20241002. doi: 10.1038/s41467-024-52721-8. 39353895 PMC11445567

[52] Vidaurre D, Smith SM, Woolrich MW. Brain network dynamics are hierarchically organized in time. Proc Natl Acad Sci U S A. 2017;114(48):12827–32. Epub 20171030. doi: 10.1073/pnas.1705120114. 29087305 PMC5715736

[53] Chishti MA, Yang DS, Janus C, Phinney AL, Horne P, Pearson J, Early-onset amyloid deposition and cognitive deficits in transgenic mice expressing a double mutant form of amyloid precursor protein 695. J Biol Chem. 2001;276(24):21562–70. doi: 10.1074/jbc.M100710200. 11279122

[54] Neuner SM, Heuer SE, Huentelman MJ, O’Connell KMS, Kaczorowski CC. Harnessing Genetic Complexity to Enhance Translatability of Alzheimer’s Disease Mouse Models: A Path toward Precision Medicine. Neuron. 2019;101(3):399–411 e5. doi: 10.1016/j.neuron.2018.11.040. 30595332 PMC6886697

[55] Colon-Perez LM, Ibanez KR, Suarez M, Torroella K, Acuna K, Ofori E, Neurite orientation dispersion and density imaging reveals white matter and hippocampal microstructure changes produced by Interleukin-6 in the TgCRND8 mouse model of amyloidosis. Neuroimage. 2019;202:116138. doi: 10.1016/j.neuroimage.2019.116138. 31472250 PMC6894485

[56] McFarland KN, Ceballos C, Rosario A, Ladd T, Moore B, Golde G, Microglia show differential transcriptomic response to Abeta peptide aggregates ex vivo and in vivo. Life Sci Alliance. 2021;4(7). Epub 20210614. doi: 10.26508/lsa.202101108. PMC8321667 34127518

[57] Oakley H, Cole SL, Logan S, Maus E, Shao P, Craft J, Intraneuronal beta-amyloid aggregates, neurodegeneration, and neuron loss in transgenic mice with five familial Alzheimer’s disease mutations: potential factors in amyloid plaque formation. J Neurosci. 2006;26(40):10129–40. doi: 10.1523/JNEUROSCI.1202-06.2006. 17021169 PMC6674618

[58] Cox RW. AFNI: software for analysis and visualization of functional magnetic resonance neuroimages. Comput Biomed Res. 1996;29(3):162–73. 8812068 10.1006/cbmr.1996.0014

[59] Jenkinson M, Beckmann CF, Behrens TE, Woolrich MW, Smith SM. Fsl. Neuroimage. 2012;62(2):782–90. doi: 10.1016/j.neuroimage.2011.09.015. 21979382

[60] Febo M, Mahar R, Rodriguez NA, Buraima J, Pompilus M, Pinto AM, Age-related differences in affective behaviors in mice: possible role of prefrontal cortical-hippocampal functional connectivity and metabolomic profiles. Front Aging Neurosci. 2024;16:1356086. Epub 20240308. doi: 10.3389/fnagi.2024.1356086. 38524115 PMC10957556

[61] Beckmann CF, Smith SM. Probabilistic independent component analysis for functional magnetic resonance imaging. IEEE Trans Med Imaging. 2004;23(2):137–52. doi: 10.1109/TMI.2003.822821. 14964560

[62] Klein A, Andersson J, Ardekani BA, Ashburner J, Avants B, Chiang MC, Evaluation of 14 nonlinear deformation algorithms applied to human brain MRI registration. Neuroimage. 2009;46(3):786–802. doi: 10.1016/j.neuroimage.2008.12.037. 19195496 PMC2747506

[63] Johnson GA, Badea A, Brandenburg J, Cofer G, Fubara B, Liu S, Waxholm space: an image-based reference for coordinating mouse brain research. Neuroimage. 2010;53(2):365–72. Epub 20100701. doi: 10.1016/j.neuroimage.2010.06.067. 20600960 PMC2930145

[64] Rubinov M, Sporns O. Complex network measures of brain connectivity: uses and interpretations. Neuroimage. 2010;52(3):1059–69. doi: 10.1016/j.neuroimage.2009.10.003. 19819337

[65] Vidaurre D. A new model for simultaneous dimensionality reduction and time-varying functional connectivity estimation. PLoS Comput Biol. 2021;17(4):e1008580. Epub 20210416. doi: 10.1371/journal.pcbi.1008580. 33861733 PMC8081334

[66] Bishop CM. Pattern recognition and machine learning. New York: Springer; 2006. xx, 738 p. p.

[67] Tsurugizawa T, Yoshimaru D. Impact of anesthesia on static and dynamic functional connectivity in mice. Neuroimage. 2021;241:118413. Epub 20210720. doi: 10.1016/j.neuroimage.2021.118413. 34293463

[68] Gutierrez-Barragan D, Singh NA, Alvino FG, Coletta L, Rocchi F, De Guzman E, Unique spatiotemporal fMRI dynamics in the awake mouse brain. Curr Biol. 2022;32(3):631–44 e6. Epub 20220107. doi: 10.1016/j.cub.2021.12.015. 34998465 PMC8837277

[69] Bukhari Q, Schroeter A, Cole DM, Rudin M. Resting State fMRI in Mice Reveals Anesthesia Specific Signatures of Brain Functional Networks and Their Interactions. Front Neural Circuits. 2017;11:5. doi: 10.3389/fncir.2017.00005. 28217085 PMC5289996

[70] Grandjean J, Preti MG, Bolton TAW, Buerge M, Seifritz E, Pryce CR, Dynamic reorganization of intrinsic functional networks in the mouse brain. Neuroimage. 2017;152:497–508. Epub 20170314. doi: 10.1016/j.neuroimage.2017.03.026. 28315459

[71] Park YG, Sohn CH, Chen R, McCue M, Yun DH, Drummond GT, Protection of tissue physicochemical properties using polyfunctional crosslinkers. Nat Biotechnol. 2018. Epub 2018/12/18. doi: 10.1038/nbt.4281. PMC6579717 30556815

[72] Yun DH, Park YG, Cho JH, Kamentsky L, Evans NB, DiNapoli N, Uniform volumetric single-cell processing for organ-scale molecular phenotyping. Nat Biotechnol. 2025. Epub 20250124. doi: 10.1038/s41587-024-02533-4. 39856430

[73] Perens J, Salinas CG, Skytte JL, Roostalu U, Dahl AB, Dyrby TB, An Optimized Mouse Brain Atlas for Automated Mapping and Quantification of Neuronal Activity Using iDISCO+ and Light Sheet Fluorescence Microscopy. Neuroinformatics. 2021;19(3):433–46. Epub 2020/10/17. doi: 10.1007/s12021-020-09490-8. 33063286 PMC8233272

[74] Goubran M, Leuze C, Hsueh B, Aswendt M, Ye L, Tian Q, Multimodal image registration and connectivity analysis for integration of connectomic data from microscopy to MRI. Nat Commun. 2019;10(1):5504. Epub 2019/12/05. doi: 10.1038/s41467-019-13374-0. 31796741 PMC6890789

[75] Vidaurre D, Masaracchia L, Larsen NY, Ruijters LRPT, Alonso S, Ahrends C, The Gaussian-linear hidden Markov model: A Python package. Imaging Neuroscience. 2024;3:1–16. doi: 10.1162/imag_a_00460. PMC12319829 40800983

[76] Schaefer A, Kong R, Gordon EM, Laumann TO, Zuo XN, Holmes AJ, Local-Global Parcellation of the Human Cerebral Cortex from Intrinsic Functional Connectivity MRI. Cereb Cortex. 2018;28(9):3095–114. doi: 10.1093/cercor/bhx179. 28981612 PMC6095216

[77] Xia M, Wang J, He Y. BrainNet Viewer: a network visualization tool for human brain connectomics. PLoS One. 2013;8(7):e68910. Epub 2013/07/19. doi: 10.1371/journal.pone.0068910. 23861951 PMC3701683

[78] Winkler AM, Ridgway GR, Webster MA, Smith SM, Nichols TE. Permutation inference for the general linear model. Neuroimage. 2014;92(100):381–97. Epub 20140211. doi: 10.1016/j.neuroimage.2014.01.060. 24530839 PMC4010955

[79] Beckmann CF, DeLuca M, Devlin JT, Smith SM. Investigations into resting-state connectivity using independent component analysis. Philos Trans R Soc Lond B Biol Sci. 2005;360(1457):1001–13. doi: 10.1098/rstb.2005.1634. 16087444 PMC1854918

[80] Shah D, Latif-Hernandez A, De Strooper B, Saito T, Saido T, Verhoye M, Spatial reversal learning defect coincides with hypersynchronous telencephalic BOLD functional connectivity in APP(NL-F/NL-F) knock-in mice. Sci Rep. 2018;8(1):6264. Epub 20180419. doi: 10.1038/s41598-018-24657-9. 29674739 PMC5908850

[81] Grandjean J, Derungs R, Kulic L, Welt T, Henkelman M, Nitsch RM, Complex interplay between brain function and structure during cerebral amyloidosis in APP transgenic mouse strains revealed by multi-parametric MRI comparison. Neuroimage. 2016;134:1–11. doi: 10.1016/j.neuroimage.2016.03.042. 27033685

[82] Bezzina C, Verret L, Juan C, Remaud J, Halley H, Rampon C, Early onset of hypersynchronous network activity and expression of a marker of chronic seizures in the Tg2576 mouse model of Alzheimer’s disease. PLoS One. 2015;10(3):e0119910. Epub 20150313. doi: 10.1371/journal.pone.0119910. 25768013 PMC4358928

[83] Busche MA, Wegmann S, Dujardin S, Commins C, Schiantarelli J, Klickstein N, Tau impairs neural circuits, dominating amyloid-beta effects, in Alzheimer models in vivo. Nat Neurosci. 2019;22(1):57–64. Epub 20181217. doi: 10.1038/s41593-018-0289-8. 30559471 PMC6560629

[84] Thompson GJ, Pan WJ, Magnuson ME, Jaeger D, Keilholz SD. Quasi-periodic patterns (QPP): large-scale dynamics in resting state fMRI that correlate with local infraslow electrical activity. Neuroimage. 2014;84:1018–31. doi: 10.1016/j.neuroimage.2013.09.029. 24071524 PMC3869452

[85] Belloy ME, Shah D, Abbas A, Kashyap A, Rossner S, Van der Linden A, Quasi-Periodic Patterns of Neural Activity improve Classification of Alzheimer’s Disease in Mice. Sci Rep. 2018;8(1):10024. Epub 20180703. doi: 10.1038/s41598-018-28237-9. 29968786 PMC6030071

[86] Benisty H, Barson D, Moberly AH, Lohani S, Tang L, Coifman RR, Rapid fluctuations in functional connectivity of cortical networks encode spontaneous behavior. Nat Neurosci. 2024;27(1):148–58. Epub 20231130. doi: 10.1038/s41593-023-01498-y. 38036743 PMC11316935

[87] Pompilus M, Colon-Perez LM, Grudny MM, Febo M. Contextual experience modifies functional connectome indices of topological strength and efficiency. Sci Rep. 2020;10(1):19843. Epub 2020/11/18. doi: 10.1038/s41598-020-76935-0. 33199790 PMC7670469

[88] Shahsavarani S, Thibodeaux DN, Xu W, Kim SH, Lodgher F, Nwokeabia C, Cortex-wide neural dynamics predict behavioral states and provide a neural basis for resting-state dynamic functional connectivity. Cell Rep. 2023;42(6):112527. Epub 20230526. doi: 10.1016/j.celrep.2023.112527. 37243588 PMC10592480

[89] Kang M, Lee YB, Gohel B, Yoo K, Lee P, Chung J, Momentary level of slow default mode network activity is associated with distinct propagation and connectivity patterns in the anesthetized mouse cortex. J Neurophysiol. 2018;119(2):441–58. Epub 20171025. doi: 10.1152/jn.00163.2017. 29070626

[90] Yee JR, Kenkel WM, Kulkarni P, Moore K, Perkeybile AM, Toddes S, BOLD fMRI in awake prairie voles: A platform for translational social and affective neuroscience. Neuroimage. 2016;138:221–32. Epub 20160527. doi: 10.1016/j.neuroimage.2016.05.046. 27238726 PMC4933013

[91] Mao H, Shi Y, Gao Q, Xu M, Hu X, Wang F, Alterations in static and dynamic functional network connectivity in subcortical vascular cognitive impairment. Sci Rep. 2025;15(1):22113. Epub 20250701. doi: 10.1038/s41598-025-06640-3. 40595059 PMC12218908

[92] Chen F, Chen Q, Zhu Y, Long C, Lu J, Jiang Y, Alterations in Dynamic Functional Connectivity in Patients with Cerebral Small Vessel Disease. Transl Stroke Res. 2024;15(3):580–90. Epub 20230327. doi: 10.1007/s12975-023-01148-2. 36967436 PMC11106163

[93] Fu Z, Caprihan A, Chen J, Du Y, Adair JC, Sui J, Altered static and dynamic functional network connectivity in Alzheimer’s disease and subcortical ischemic vascular disease: shared and specific brain connectivity abnormalities. Hum Brain Mapp. 2019;40(11):3203–21. Epub 20190405. doi: 10.1002/hbm.24591. 30950567 PMC6865624

[94] Daley L, Pan WJ, Kaundinya G, Keilholz S. Longitudinal Awake Mouse fMRI During Voluntary Locomotion Using Zero TE Imaging and a Novel Treadmill Training Protocol. Magn Reson Med. 2026;95(5):2840–51. Epub 20260108. doi: 10.1002/mrm.70248. 41505251 PMC12962197

