# Supplementary material for: Dissociable species-specific impact of Aβ on static and dynamic functional connectomes
Source: bioRxiv. 2026 Apr 29:2026.04.26.720907. Preprint. [Version 1] doi: 10.64898/2026.04.26.720907 (PMC13142524; doi:10.64898/2026.04.26.720907)
Supplement: Supplement 1 [file NIHPP2026.04.26.720907v1-supplement-1.pdf]

bioRxiv preprint doi: https://doi.org/10.64898/2026.04.26.720907; this version posted April 29, 2026. The copyright holder for this preprint (which was not certified by peer review) is the author/funder, who has granted bioRxiv a license to display the preprint in perpetuity. It is made available under aCC-BY 4.0 International license.

Supporting Information

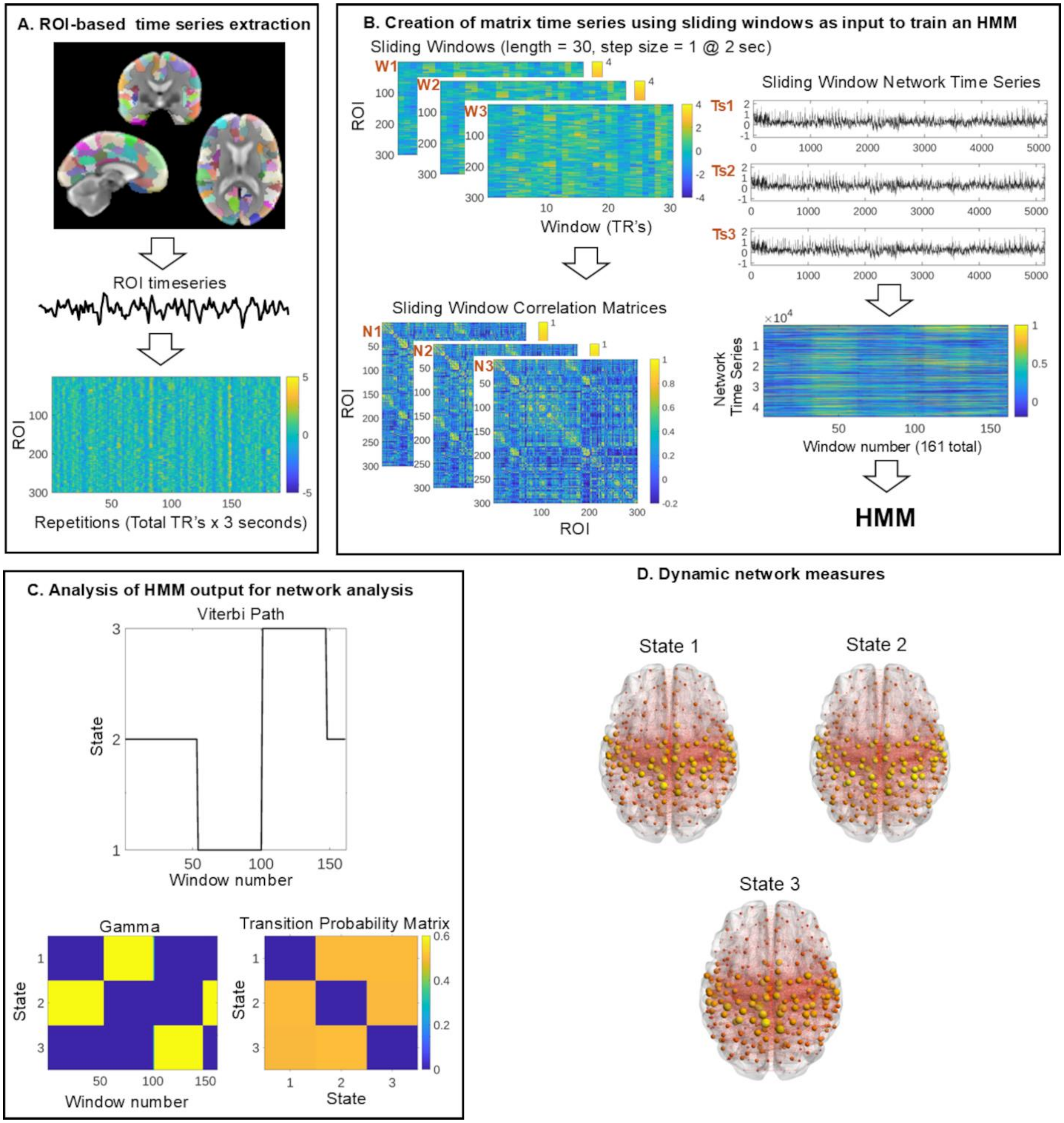

Fig S1. Processing of ADNI data-derived fMRI signals for HMM-MAR. A) Signals were extracted from post-processed images aligned to the Shaeffer 300 parcellation[63]. B) Following a sliding window, network matrices were constructed per window and vectorized and organized in a network x window matrix as input to HMM-MAR[75]. C) HMM outputs. D) Network visualizations across states.

bioRxiv preprint doi: https://doi.org/10.64898/2026.04.26.720907; this version posted April 29, 2026. The copyright holder for this preprint (which was not certified by peer review) is the author/funder, who has granted bioRxiv a license to display the preprint in perpetuity. It is made available under aCC-BY 4.0 International license.

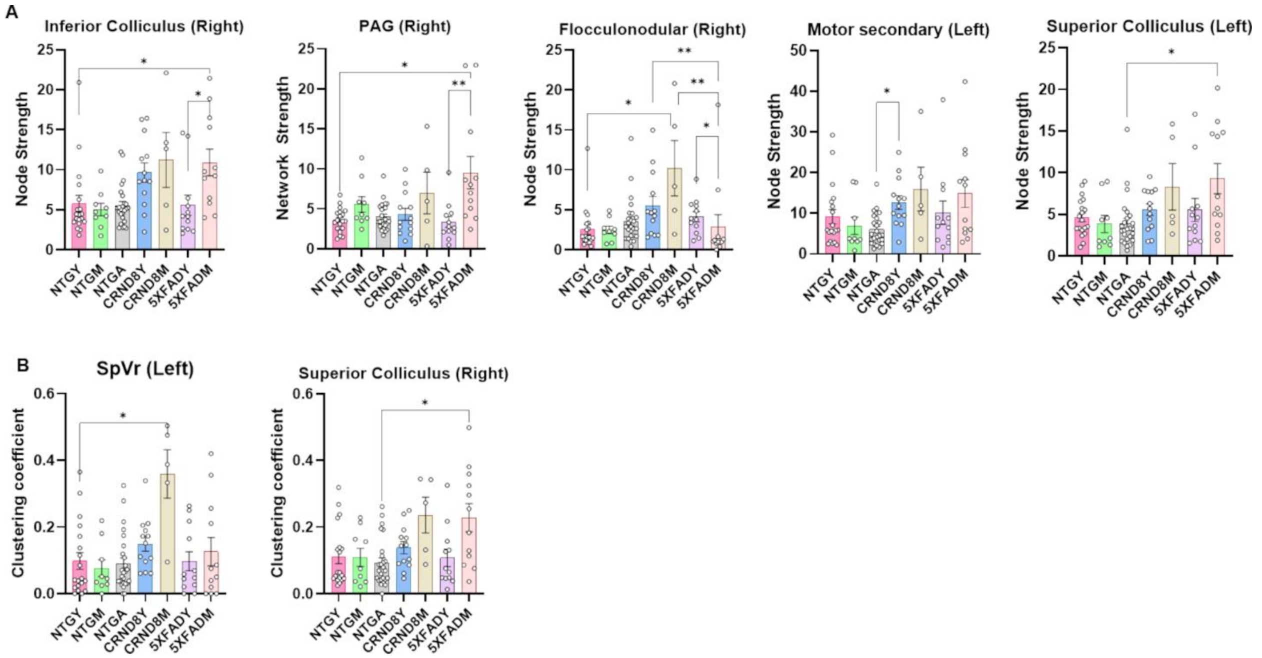

Fig S2. Node strength and clustering coefficient in additional brain areas affected by amyloidosis. Groups are as shown in Figure 2. A) Node strength. B) Clustering coefficient. All data presented as mean ± standard error with overlaid scatter plots. Significant differences tested across all nodes using linear mixed effects ANOVA (FDR corrected). Post hoc Dunn's tests indicated by asterisks (*p<0.05, **p<0.01).
